# Selection and evolution of disulfide-rich peptides *via* cellular protein quality control†

**DOI:** 10.1039/d2sc05343h

**Published:** 2023-03-15

**Authors:** Xiaoting Meng, Chaoying Xu, Shihui Fan, Meng Dong, Jie Zhuang, Zengping Duan, Yibing Zhao, Chuanliu Wu

**Affiliations:** a Department of Chemistry, College of Chemistry and Chemical Engineering, The MOE Key Laboratory of Spectrochemical Analysis and Instrumentation, State Key Laboratory of Physical Chemistry of Solid Surfaces, Xiamen University Xiamen 361005 P. R. China chlwu@xmu.edu.cn

## Abstract

Disulfide-rich peptides (DRPs) are an interesting and promising molecular format for drug discovery and development. However, the engineering and application of DRPs rely on the foldability of the peptides into specific structures with correct disulfide pairing, which strongly hinders the development of designed DRPs with randomly encoded sequences. Design or discovery of new DRPs with robust foldability would provide valuable scaffolds for developing peptide-based probes or therapeutics. Herein we report a cell-based selection system leveraging cellular protein quality control (termed PQC-select) to select DRPs with robust foldability from random sequences. By correlating the foldability of DRPs with their expression levels on the cell surface, thousands of sequences that can fold properly have been successfully identified. We anticipated that PQC-select will be applicable to many other designed DRP scaffolds in which the disulfide frameworks and/or the disulfide-directing motifs can be varied, enabling the generation of a variety of foldable DRPs with new structures and superior potential for further developments.

## Abstract 2

A selection system leveraging cellular protein quality control (termed PQC-select) has been designed to select DRPs with robust foldability from random sequences, providing valuable scaffolds for developing peptide-based probes or therapeutics.

## Graphical abstract

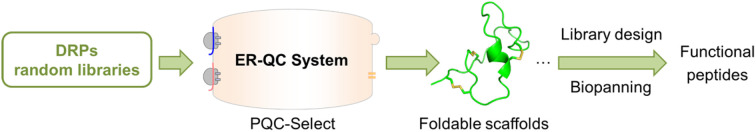

## Introduction

Disulfide-rich peptides (DRPs) evolved by natural selection over billions of years have attracted immense interest for the development of new therapeutics.^1–4^ These DRPs usually have stable protein-like structures that are crucial for their target-binding specificity and potency and high proteolytic stability.^5–7^ By exploiting these DRPs as templates, new structures and functions can also be acquired through epitope grafting and sequence evolution.^8–11^ However, the development of new DRPs is significantly hindered by the limited variety in structures of the naturally occurring DRP scaffolds. Efforts to address this issue mainly focus on the development of new methods to *de novo* design DRP scaffolds, including computational methods to design structurally ordered DRPs and our approaches relying on the orthogonal disulfide-directing motifs to design DRPs with varied patterns of thiol-bearing amino acids.^12–18^ However, given the immense sequence space of the peptides with varied lengths and amino acid patterns, it remains a challenge to identify new DRPs with robust foldability from random sequences. Designed DRPs with the ability to fold like natural DRPs can provide scaffolds to generate functional peptides useful to biology or therapeutics through directed evolution or epitope grafting.

Foldability is an intrinsic property of most natural proteins and DRPs, which is the result of synergy between natural evolution and cellular protein quality control to generate functional polypeptides with well-defined 3D structures that are determined by primary amino acid sequences.^19–24^ Particularly, for the secreted and cell-surface proteins, sequences that are able to afford foldability can be selected through evolution and produced by cellular protein expression and folding systems.^25–28^ In eukaryotic cells, protein quality control predominantly takes place in the endoplasmic reticulum, where the proteins fold properly and are subsequently exported, and the misfolded polypeptides are subjected to degradation by the ubiquitin-proteasome system.^29–33^ This mechanism to monitor protein folding has been exploited for the expression and cell-surface display of proteins or DRPs (both naturally derived and *de novo* designed) that are difficult to be produced by prokaryotic cells, as the protein quality control in prokaryotic cells is significantly less stringent.^34–36^ Undoubtedly, mammalian cells possess a most sophisticated and rigorous protein quality control system, which enables the production and secretion of a wide variety of proteins with well-defined disulfide-rich segments. This specialty has recently been exploited for the construction of DRP libraries derived from both naturally occurring and computationally designed folds.^34,37^ Nevertheless, the protein quality control of mammalian cells has not been exploited for the selection of *de novo* DRPs from random sequences. Novel DRPs identified by leveraging cellular protein quality control would exhibit robust foldability like natural proteins, representing promising scaffolds for further developments.

Herein, we report the design and construction of a cellular protein quality control-based selection (PQC-select) system for identifying *de novo* DRPs from random sequences. By taking advantage of the protein quality control in mammalian cells, random sequences with robust foldability like natural DRPs can be exported from the endoplasmic reticulum to the cell surface, whereas those that are unable to fold properly are subjected to degradation. Thus, the foldability of DRPs can be correlated with their expression levels on the cell surface, enabling rapid and efficient selection of DRPs from random sequences. PQC-select provides a novel, convenient and efficient strategy to generate novel DRPs with robust foldability, and high cell surface display efficiency and potential in developing peptide therapeutics.

Recently, we have developed a wide variety of new DRPs by incorporating CXC and/or CPPC motifs (C: cysteine; X: any residues; P: proline) into random sequences.^15–18^ Though several DRPs with well-defined 3D structures have been obtained from the screening of libraries against protein targets,^17^ it remains unclear if these peptides, as a new class of artificial DRPs, can pass cellular protein quality control like natural proteins. This will be important for biological applications involving display of functional DRPs on the cell surface such as in engineered cell-based therapies and for their further development involving mammalian cell-based directed evolution. Compared to bacteria and yeasts, mammalian cells have evolved more complex secretory pathways to manipulate oxidative folding of proteins with disulfide-rich segments.^34^ In this work, we set out to examine the compatibility of the oxidative folding of DRPs with two CPPC motifs and the folding machinery in the endoplasmic reticulum, and aim to leverage the protein quality control system of mammalian cells to develop PQC-select for identifying novel CPPC-DRPs with foldability in cells from random sequences.

## Results and discussion

**Fig. 1:**
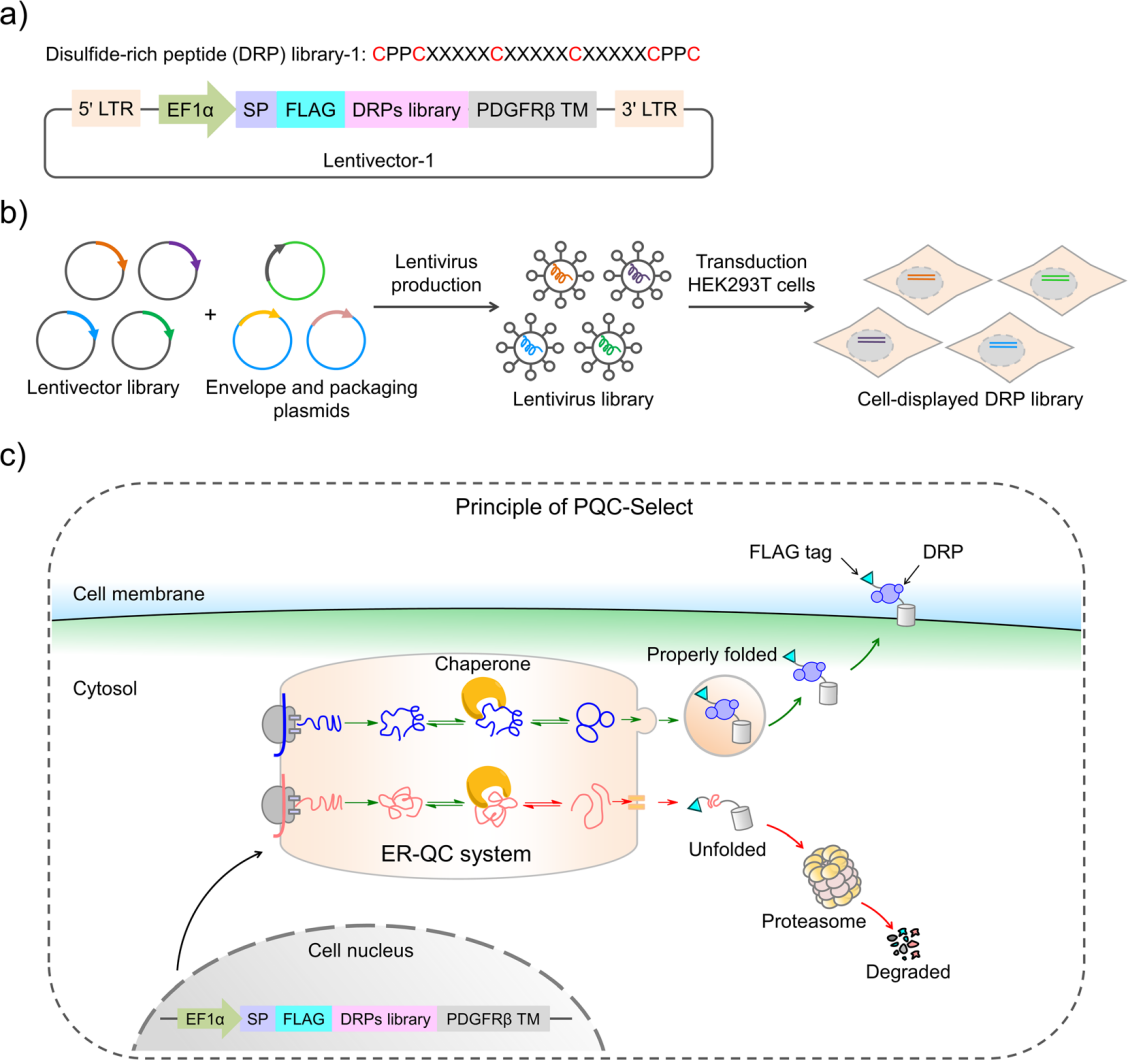
Design and principle of the PQC-select strategy. (a) Construction of a cell-displayed DRP lentivector-1 (5′ LTR: 5′ a long terminal repeat, 3′ LTR: 3′ a long terminal repeat, EF1α: a promotor, SP: a signal peptide (the murine Igκ-chain leader sequence), FLAG: an epitope tag, and PDGFRβ TM: the platelet-derived growth factor receptor beta transmembrane domain). The corresponding oligonucleotides of DRPs were synthesized, amplified, and then cloned into the lentivector to produce a lentivector library-1. (b) A schematic to show lentivirus production and transduction into HEK293T cells. The lentivirus library was produced in HEK293T cells by co-transfecting the lentivector library with envelope (pMD2.G-VSV) and packaging plasmids (pMDLg and pRSV-REWV), which was then transduced into HEK293T cells to produce a cell-displayed DRP library. (c) A schematic to show the principle of PQC-select. An exogenous gene among 5′ LTR and 3′ LTR was integrated into the genomes of the transduced HEK293T cells. The signal sequence would direct the translocation of DRPs into the endoplasmic reticulum (ER) where they fold, and disulfide bonds were formed which rely on chaperon systems, and finally the properly folded DRPs would be transported to the cell surface with the assistance of the C-terminal transmembrane domain and tracked through fluorescent staining of the FLAG tag. When DRPs were unfolded or subjected to aggregations, they would be retained in the ER and translated back to the cytosol for degradation by the proteasomes. ER-QC: endoplasmic reticulum quality control.

Construction of PQC-select involves the generation of a lentivirus library with a lentivector containing random sequences flanked by a signal peptide from the murine Igκ-chain leader sequence, a FLAG tag at the N-terminus and a transmembrane domain from platelet-derived growth factor receptor beta (PDGFRβ) at the C-terminus, transduction of mammalian cells with the lentivirus library, expression and display of folded peptides on the cell surface, and fluorescent staining and selection of cells with high peptide display levels using flow cytometry (Fig. 1). Random peptides consist of two CPPC and two cysteines isolated by three five-residue random segments. The signal sequence directs the translocation of peptides into the endoplasmic reticulum where they fold and disulfide bonds are formed, and finally the properly folded DRPs will be transported to the cell surface with the assistance of the C-terminal transmembrane domain. We speculated that sequences that are unable to fold properly or subjected to aggregations will be translocated back into the cytosol for degradation, and sequences with robust foldability can be efficiently displayed on the cell surface and tracked through fluorescent staining of the FLAG tag.

**Fig. 2:**
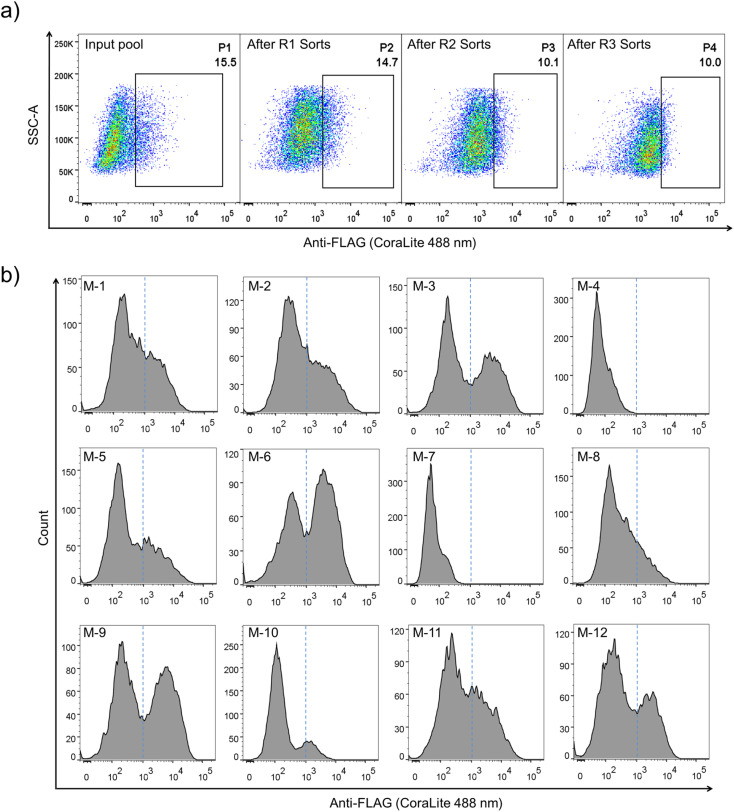
Selection and identification of cell-displayed DRP library-1. (a) FACS progressions of cell-displayed DRP library-1; shown are the profiles of the unsorted input library (input pool), and the library after one (after R1 sorts), two (After R2 sorts), or three (after R3 sorts) sorts. The cell population circled in the box was sorted at each round and amplified for the next round of sorting. Four rounds of FACS were used to obtain an enriched cell population (P4) with extremely high surface-displayed levels of DRPs. (b) Immunofluorescence flow cytometry analysis of cell-displayed efficiency of 12 randomly selected DRPs from the P4 cell population.

The lentivector library (size: 3.0 × 10^6^ pfu) was first constructed through DNA cloning and transformation of *E. coli*. Then, the lentivirus library was produced by co-transfection of the lentivector library with envelope and packaging plasmids in HEK293T cells, and the lentiviral titer was determined using qPCR. HEK293T cells (2.8 × 10^7^ cells) were then transduced with the lentivirus library (1.9 × 10^7^ TU) at a MOI (multiplicity of infection) of ∼0.7. After ∼68 h of incubation, the transduced cells were harvested, stained fluorescently, and analyzed using flow cytometry (FACS; Fig. 1 and 2a). A substantial number of the transduced cells exhibit very low fluorescence emission comparable to that observed from cells without the virus infection (blank) and cells with the infection of viruses without the inserted random peptide and FLAG tag (negative control), suggesting that a number of random sequences cannot be folded properly and displayed on the cell surface (Fig. 2a and S1†). However, there are still transduced cells with high fluorescence intensity, indicating high expression and displaying levels of DRPs. These cells (∼15.5% of total cells) were collected and subsequently subjected to four iterative cycles of culture and sorting to obtain cells with extremely high surface-displayed levels of DRPs. Indeed, we observed a gradual increase in the cell fluorescence after each sorting cycle (Fig. 2a). The resulting proportion of cells which represents 0.23‰ of the initial total input (*i.e.*, the product of the four sorting fractions) exhibits a median fluorescence intensity (MFI) that is one to two orders of magnitude higher than that from the initial unsorted cells. We sequenced the DRPs selected from the iterative sorting using next-generation sequencing. Sequences in line with the designed cysteine pattern without additional cysteine(s) were analyzed and arranged based on the abundance normalized to that found in the initial sequence pool (dataset S1). A total of ∼2217 different sequences with high diversity were obtained. Despite this, some sequences with stop codon(s) were also enriched, suggesting the presence of sequences escaping protein quality control (dataset S2). Thus, we further deep-sequenced 10 individual cell clones isolated by FACS, and found that there are 3–8 different sequences in each cell clone (Fig. S3†). It seems that cells infected simultaneously by multiple lentiviruses are preferentially enriched during the selection, as these cells are more capable of producing high levels of DRPs. Though there are sequences escaping protein quality control enrichment, further examination of 12 random individual clones among the abundantly enriched sequences shows that 10 of them (∼80%) can be efficiently expressed and displayed on the cell surface after lentiviral infection (Fig. 2b and S4†), indicating the success of the PQC-select strategy.

**Fig. 3:**
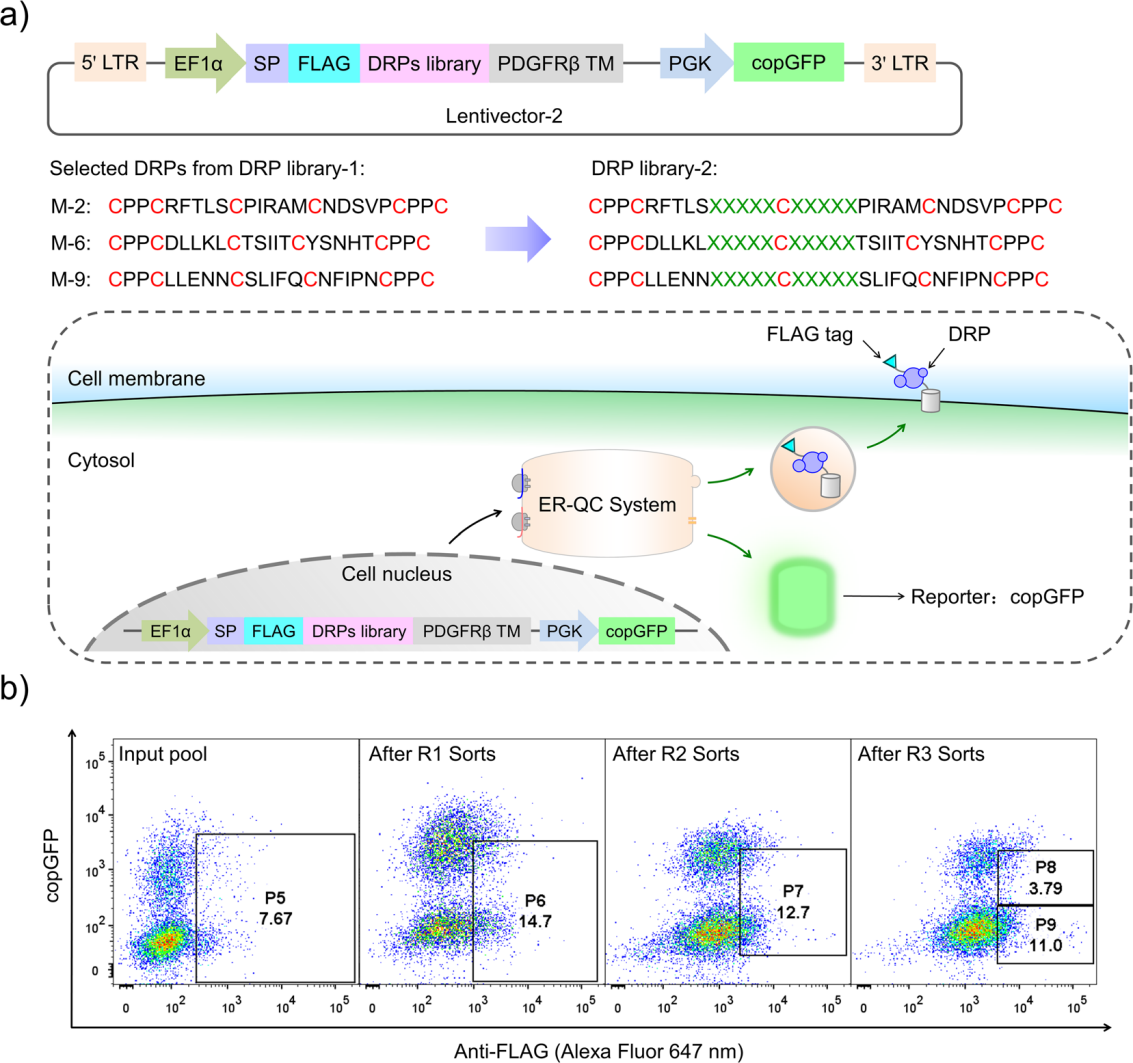
Design and selection of novel CPPC-DRPs. (a) Construction of a cell-displayed DRP lentivector-2. PGK: a promotor and copGFP: a fluorescent protein reporter. The corresponding oligonucleotides of novel CPPC-DRPs by incorporating ten consecutive random residues into three DRPs selected from DRP library-1 were synthesized, amplified, and then cloned into the cell-displayed DRP lentivector-2 to produce a lentiviral plasmid library-2. A schematic to show the principle of the second-generation PQC-select. (b) FACS progressions (copGFP *vs.* Anti-FLAG-Alexa Fluor 647 nm) of cell-displayed DRP library-2; shown are the profiles of the unsorted input library (input pool), and the library after one (after R1 sorts), two (after R2 sorts), or three (after R3 sorts) sorts. The cell population circled in the boassorted at each round and amplified for the next round of sorting. Four rounds of FACS were used to obtain an enriched cell population (P8 and P9) with extremely high surface-displayed levels of novel CPPC-DRPs.

Although successful, lessons learned from the previous exploration motivated us to redesign the DRP display construct of the PQC-select to avoid extensive enrichment of sequences escaping protein quality control. By incorporating a copGFP (a green fluorescent protein cloned from copepod pontellina plumata) reporter into the lentivector, cells infected by multiple lentiviruses might be excluded in FACS by setting a proper threshold for copGFP signals (Fig. 3a and S2†). Instead of reselecting the previous CPPC-DRPs using the new DRP-select, we designed a new library by incorporating ten consecutive random residues into three DRPs with robust foldability selected previously, aiming to identify novel CPPC-DRPs with longer sequences. We argue that larger DRPs are more prone to suffering from folding problems, and DRPs identified previously are a good starting point for sequence space expansion. By following the same procedures as before, transduced cells analyzed by FACS show two separate copGFP fluorescence populations, indicating cells with relatively higher and lower copGFP expression, respectively. The cells were then iteratively selected four times by sorting the populations with lower copGFP and higher surface FLAG-stain fluorescence (Fig. 3b). The threshold of copGFP fluorescence for the selection was set to include both the higher and lower copGFP-expression populations to enable further comparison. The two separate cell populations were finally sorted and analyzed, respectively. Deep-sequencing of randomly selected cell clones (15 clones) in the higher GFP-expression population (P8) shows that most clones contain two or more different DRP sequences (13 clones; Fig. S5†). In contrast, most cells in the lower copGFP-expression population (P9) have only one DRP sequence (20 amongst 26 clones; Fig. S6†), suggesting the success of using copGFP as a reporter to exclude cells with multiple infections.

**Fig. 4:**
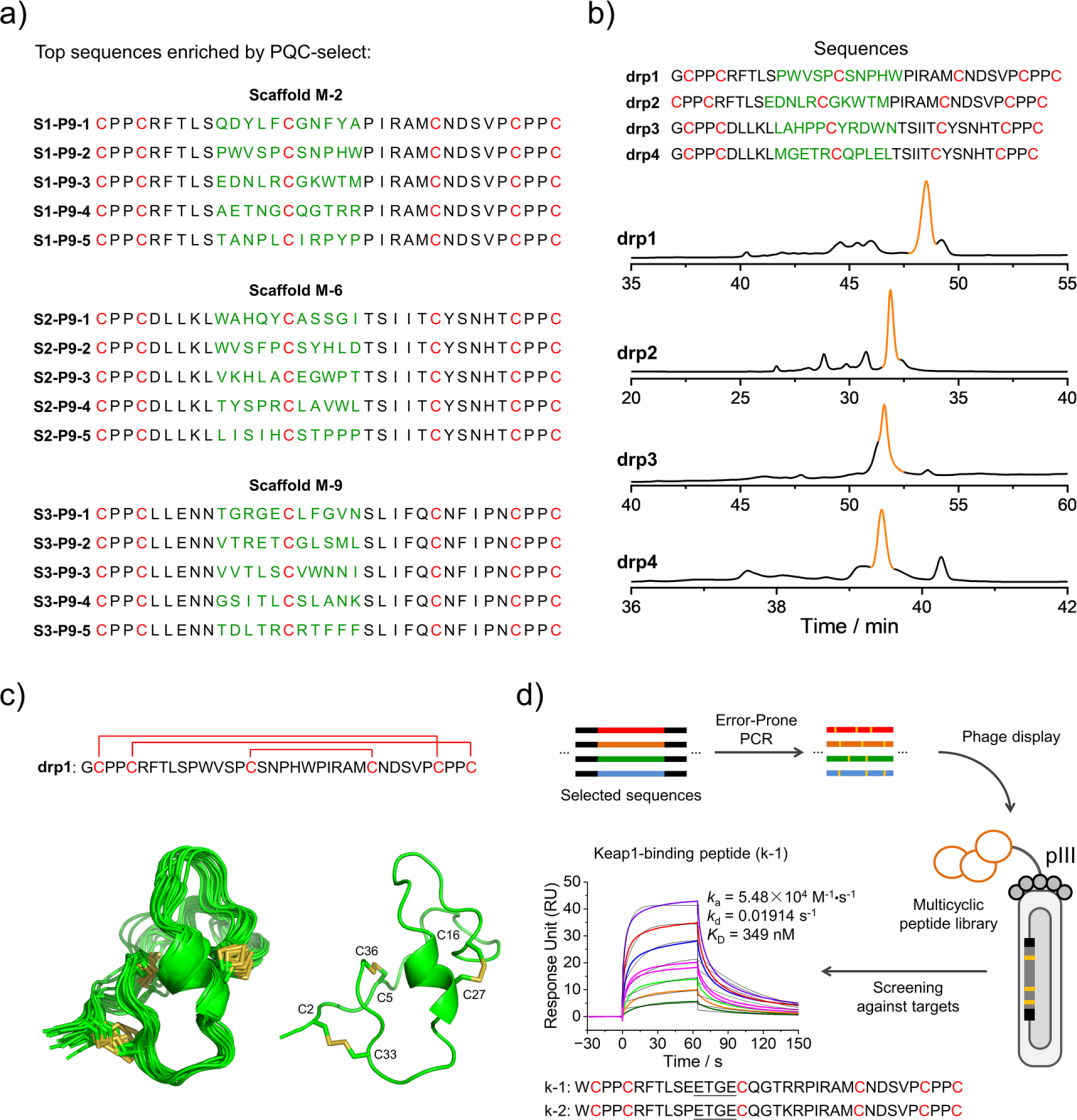
Identification and application of new DRPs selected from the second-generation PQC-select. (a) Top 5 enriched sequences for the three different scaffolds obtained from the second-generation PQC-select, scaffold M-2 (top), scaffold M-6 (middle) and scaffold M-9 (bottom). (b) HPLC chromatograms showing the oxidation of drp1, drp2, drp3 and drp4 in phosphate buffers (pH 7.4, 100 mM) containing 0.2 mM GSSG or 6 M Gu·HCl and 0.2 mM GSSG. (c) Solution NMR structures of drp1. (Left: ensemble of the 15 lowest-energy structures; right: cartoon depiction of the lowest-energy structure; PDB 8GUC). (d) Schematic view of constructing a phage-displayed DRP library through the error-prone PCR using the sequences selected by the PQC-select as a precursor pool to generate new DRPs with Keap1-binding capability. SPR sensorgrams showing interaction of Keap1 with the oxidized products of k-1 in a concentration-dependent manner, and the equilibrium dissociation constant (*K*_D_) values of k-1 toward Keap1 calculated from SPR.

We then deep-sequenced the DRPs enriched in P9, and a total of ∼1404 different sequences with the expected cysteine patterns were obtained (3 different scaffolds: 256, 742 and 406, respectively; dataset S3 and Fig. 4a). Sequences with stop codon(s) are very rare compared to that found in the previous selection (dataset S4), further confirming the success of the second-generation PQC-select. These results thus demonstrated the usefulness of PQC-select in generating *de novo* CPPC-DRPs that can express and fold in cellular environments, providing a large number of new DRP templates for further developments. To chemically examine the foldability of the selected DRPs, four sequences were randomly selected for synthesis and oxidation. All of them can be specifically oxidized to a major product as determined using HPLC (Fig. 4b and S7†). We further characterized solution structures of one of the DRPs (drp1) using NMR. ^1^H–^1^H TOCSY spectra of the peptide show good dispersion, illustrating that the peptide was well-folded in solution. The disulfide pairing of drp1 was clearly shown by the ^1^H–^1^H NOESY spectra (Fig. S10†). The 3D structures indicate the formation of irregular but rigid loops and a short α-helix constrained through three disulfide bonds with the two CPPC motifs paired parallelly (Fig. 4c).

We believe that the DRPs identified through the PQC-select can be useful scaffolds for further developments. For example, functional peptides might be developed by combining epitope grafting, library construction and screening, as described previously.^17^ Alternatively, the selected sequences might serve as a precursor pool for sequence diversification through the error-prone PCR to create peptide libraries, using which ligands to target proteins might be selected (Fig. 4d). We argued that the foldability of these PQC-selected sequences will not be significantly affected by a few mutations introduced by the error-prone PCR, and thus they can be efficiently displayed using a phage display system without significant changes in their overall structures and foldability. As a proof-of-concept, we constructed a phage-display peptide library by inserting the PCR products into phagemid vectors, and a model protein (Keap1) was used as a target for the screening application.^38^ We observed extensive enrichment of phages after three rounds of selection, suggesting the successful discovery of Keap1-binding peptides from the library. Phage clones were then randomly picked for Sanger sequencing, and two sequences containing a typical Keap1-binding motif ETGE were obtained (Fig. 4d).^39^ In comparison with sequences enriched in P9, we found that these two peptides (k-1 and k-2) were obtained from the error-prone PCR of S1–P9-4 (*i.e.*, one of the top five sequences enriched from scaffold M-2). k-1 and k-2 were then synthesized chemically, which can be oxidized to a major product that can be isolated using HPLC (Fig. S8 and S11†). Binding affinities of these two oxidized peptides (the major oxidized product of k-1 and k-2) to Keap1 were then measured using surface plasma resonance (SPR), revealing affinities in the submicromolar range (Fig. 4d and S11†). These results, though preliminary, demonstrated the applicability of the PQC-selected random sequences for discovering new protein binders. We anticipated that more functional peptides will be generated in the future by taking our PQC-selected DRPs as scaffolds.

## Conclusions

In summary, we have developed a mammalian cell-based selection system (called PQC-select) for the discovery of *de novo* DRPs with robust foldability, which exploits cellular protein quality control for selection. By correlating the foldability of DRPs with their expression levels on the cell surface, thousands of sequences containing two CPPC motifs and two isolated cysteine residues that can fold properly have been successfully identified. In principle, PQC-select should be applicable to other disulfide-directed peptide scaffolds where the disulfide frameworks and/or the disulfide-directing motifs (*e.g.*, CPPC and CXC) can be varied, which would enable the generation of a variety of foldable DRPs with new structures. One challenge moving forward will be the characterization of accurate structures for so many peptide sequences identified from the selection in this work and any other selections in the future. However, this might be addressed hopefully by the further development in artificial intelligence for predicting protein folding.^40^ Compared to other display systems such as phages and yeast, though mammalian cell display systems usually have smaller library capacity, mammalian cells possess rigorous protein quality control to enable surface display of DRPs with robust foldability and well-defined structures. This makes the mammalian cell display systems particularly suitable for the selection of foldable and rigid scaffolds like naturally occurring DRPs, instead of for directly selecting peptide binders to specific targets. However, with the discovery of these *de novo* DRPs, phage or yeast display libraries with a larger size relative to mammalian cell display libraries can be routinely developed by using these DRPs as templates, enabling the selection of new peptide binders to a variety of protein targets. We believe that the discovery of new foldable DRPs would provide the basis for the development of multicyclic peptide-based molecular probes or therapeutics.

## Data availability

The NMR structures are available in the Protein Data Bank. Other materials may be requested from the corresponding authors.

## Author contributions

X. M. and C. X. contributed equally to this work. X. M., and C. W. designed the research. X. M., C. X. and M. D. performed the experiments on cell-displayed DRP library design, construction, and screening. X. M. and C. X. performed the experiments on peptide synthesis. S. F. characterized the peptide structures. J. Z. and X. M. performed the experiments on protein expression. Z. D. and X. M. performed affinity experiments of the peptides. X. M., C. X. and C. W. analyzed the data. X. M., Y. Z. and C. W. wrote and revised the manuscript. C. W. supervised the research. All authors reviewed and approved the manuscript.

## Conflicts of interest

There are no conflicts to declare.

## Supplementary Material

SC-014-D2SC05343H-s001

SC-014-D2SC05343H-s002

SC-014-D2SC05343H-s003

SC-014-D2SC05343H-s004

SC-014-D2SC05343H-s005
