# Supplementary material for: Selection and evolution of disulfide-rich peptides via cellular protein quality control
Source: Chem Sci. 2023 Mar 15;14(13):3668–75. doi: 10.1039/d2sc05343h (PMC10055976; doi:10.1039/d2sc05343h)
Supplement: SC-014-D2SC05343H-s005 [file SC-014-D2SC05343H-s005.pdf]

Electronic Supplementary Material (ESI) for Chemical Science.
This journal is © The Royal Society of Chemistry 2023

Electronic Supplementary Information

Selection and evolution of disulfide-rich peptides via cellular protein quality control

Xiaoting Meng,‡ Chaoying Xu,‡ Shihui Fan, Meng Dong, Jie Zhuang, Zengping Duan, Yibing Zhao, and Chuanliu Wu*

The MOE Key Laboratory of Spectrochemical Analysis and Instrumentation, State Key Laboratory of Physical Chemistry of Solid Surfaces, Department of Chemistry, College of Chemistry and Chemical Engineering, Xiamen University, Xiamen, 361005, P.R. China.

*To whom correspondence should be addressed, Email: chlwu@xmu.edu.cn

List of Supplementary Material

Experimental section

Table S1. Information of cell-displayed DRP libraries

Table S2. Information of primers

Table S3. Information of peptides

Figure S1-S2. Immunofluorescence flow cytometry analysis

Figure S3-S6. Deep-sequencing and Sanger sequencing results

Figure S7-S8. Chromatograms of peptides

Figure S9. Mass spectrum of peptides

Figure S10. NMR characterization of oxidized **drp1**

Figure S11. Characterization and affinity experiments of the major oxidized product of peptides **k-1** and **k-2**

Experimental Section

Materials

Ampicillin (Amp), kanamycin (Kana), agar, bovine serum albumin (BSA), phosphate buffered saline (1×PBS), 4S Red Plus Nucleic acid dye, Acryl Carrier and Agarose were bought from Sangon Biotechnology (Shanghai, China). Lipofectamine 2000, GeneRuler 1 kb Plus DNA Ladder, *MluI*, *Bsp1407I*, *NheI*, *XbaI*, *BamHI*, Alkaline phosphatase, 0.25% trypsin-EDTA, DMEM, Opti-MEM, EZ-Link™ Sulfo-NHS-LC-Biotin, Dynabeads™ M-280 Streptavidin, Dynabeads™ M-280 Tosyl-activated and neutravidin were purchased from Thermo Fisher Scientific (Shanghai, China). Easy Dilution, RNase-free water, Recombinant DNaseI, PrimeSTAR® HS DNA Polymerase, Klenow fragment, *SfiI*, *NotI*, T4 DNA Ligase and 20 bp DNA Ladder were bought from Takara (Beijing, China). Non-enzyme Cell Detach Solution, NEBuilder® HiFi DNA Assembly Master Mix (New England Biolabs, #E2621S) and QIAprep spin Miniprep Kit (QIAGEN, #27106) were supplied by Taijing Biological (Xiamen, China). Polybrene and DNA extract solution were purchased from Solarbio (Beijing, China). Lentivirus concentration reagent, CoraLite488–conjugated Affinipure Goat Anti-Mouse IgG(H+L) (Proteintech, #SA00013-1) and Monoclonal ANTI-FLAG® M2 antibody produced in mouse (Sigma, #F1804) were supplied by Yeasen Biotechnology (Shanghai, China). Goat Anti-Mouse IgG H&L (Alexa Fluor® 647) was purchased from Abcam (Shanghai, China). SYBR® Green Premix Pro Taq HS qPCR Kit and SteadyPure Universal Genomic DNA Extraction Kit were purchased from Accurate Biotechnology (Changsha, China). Low fidelity Taq DNA polymerase was purchase from Acmec Biochemical (Shanghai, China). Plasmid pCDH-EF1α-MCS2-PGK-copGFP-T2A-Puro was bought from Hedgebio (Shanghai, China). Primers for constructing the libraries and sequencing were purchased from Genscript (Nanjing, China). Plasmids pLV CS2.0 C-flag, and pMD2.G-VSV (addgene 12259), pMDLg (addgene 12251) and pRSV-REWV (addgene 12253) were generous gifts from Prof. Jiahuai Han. HEK293T Cell and Stabl3 *E. coli* strain were generous gifts respectively from Dr. Chuanqi Zhong and Dr. Yuhsuan Tsai. TG1 *E. coli* strain was purchased from Beyotime (Shanghai, China). The designed plasmids, sequencing primers and primers for constructing the libraries were purchased from Genscript (Nanjing, China) or Tsingke Biotechnology (Tianjing, China). Fmoc-protected amino acids and MBHA resins used in peptide synthesis were purchased from GL Biochem (Shanghai, China). N, N'-Diisopropylcarbodiimide (DIC) and Acetonitrile (ACN), trifluoroacetic acid (TFA), tris (2-carboxyethyl) phosphine hydrochloride (TCEP) and glutathione oxidized (GSSG) were purchased from Sigma-Aldrich (Shanghai, China). Acetonitrile-D3 was purchased from Cambridge Isotope Laboratories (Beijing, China). Deuterium oxide (D_2O) was obtained from Energy Chemical (Shanghai, China). All chemicals were purchased commercially and used without

further purification. Millipore ultrapure water was used throughout the experiments.

Instruments

Fluorescent intensity in cells was analyzed by flow cytometry (BD FACS Aria III). A real-time PCR detection system (Bio-rad cfx96) was used to identify the designed lentivirus library titers. A CEM Discover Liberty BLUE microwave-assisted peptide synthesizer was used for synthesizing peptides. All peptides were purified and characterized by a SHIMADZU HPLC system equipped with a prominence LC-20AD solvent delivery unit, a prominence DGU-20A3R degassing unit, a prominence SIL-20A autosampler, a prominence CTO-20A column oven and a prominence SPD-M20A photodiode array detector. Chromatograms were recorded by using an Inertsil ODS-SP C18 (5 mm, 4.6×250 mm) column by using gradients of water+0.1% trifluoroacetic acid (TFA) and acetonitrile (ACN)+0.1% TFA at a flow rate of 1 mL min^{-1}. A Hitachi U-3900H UV/Vis spectrometer was used for measuring the concentrations of peptides. A Bruker autoflex max MALDI-TOF mass spectrometry was applied to identify the formed peptides. NMR experiments were recorded at 302 K on Bruker AVANCE III 600 MHz and 850 MHz equipped with a cryogenic triple-resonance probe. Fluorescence polarization was recorded by Tecan Infinite® 200 PRO Microplate Reader. Fast Protein Liquid Chromatography (FPLC) equipped with a HitrapTM Desalting column (GE Healthcare) was used to purify the expressed proteins or the biotinylated proteins. Surface Plasmon Resonance (SPR) assays were performed by Biacore T200.

Cell-displayed lentiviral vector construction and cloning

A gene encoding the murine Igκ-chain leader sequence at N-terminal followed by a GGS spacer, a FLAG tag (DYKDDDDK), a GGSGGS spacer, a TRASKLY spacer (the DNA fragment containing *MluI* and *Bsp1407I* restriction enzymes for inserting the encoded DRP random libraries), a SGSGGSGGSGGS spacer and the platelet derived growth factor receptor transmembrane domain (PDGFR TM) at C-terminal was purchased from Genscript (Nanjing, China). The insert sequence (SP-flag-PDFGRβ TM) was cloned into plasmid pLV CS2.0 C-flag using *BamHI* and *NheI* to afford pLV SP-flag-PDFGRβ TM. To construct pLV SP-flag-PDFGRβ TM-copGFP, a fragment containing copGFP gene fragment (copGFP: a green fluorescent protein cloned from copepod Pontellina plumate, copGFP was characterized by superbright green fluorescence) was amplified with primers 1 and 2 using pCDH-EF1α-MCS2-PGK-copGFP-T2A-Puro as the template and introduced by Gibson assembly into *NheI* and *SalI* digested pLV SP-flag-PDFGRβ TM fragment and verified by sequencing.

Sequence of pLV SP-flag-PDFGRβ TM

AAGCTTAATGTAGTCTTATGCAATACTCTTGTAGTCTTGCAACATGGTAACGATGAGTTAGCAACATGCC
TTACAAGGAGAGAAAAAGCACCGTGCATGCCGATTGGTGGAAGTAAGGTGGTACGATCGTGCCTTATT
AGGAAGGCAACAGACGGGTCTGACATGGATTGGACGAACCACTGAATTGCCGCATTGCAGAGATATTG
TATTTAAGTGCCTAGCTCGATACATAAACGGGTCTCTCTGGTTAGACCAGATCTGAGCCTGGGAGCTCT
CTGGCTAACTAGGGAACCCACTGCTTAAGCCTCAATAAAGCTTGCCTTGAGTGCTTCAAGTAGTGTGTG
CCCGTCTGTTGTGTGACTCTGGTAACTAGAGATCCCTCAGACCCTTTTAGTCAGTGTGGAAAATCTCTA
GCAGTGGCGCCCGAACAGGGACTTGAAAGCGAAAGGGAAACCAGAGGAGCTCTCTCGACGCAGGAC
TCGGCTTGCTGAAGCGCGCACGGCAAGAGGCGAGGGGCGGCGACTGGTGAGTACGCCAAAAATTTTG
ACTAGCGGAGGCTAGAAGGAGAGAGATGGGTGCGAGAGCGTCAGTATTAAGCGGGGGAGAATTAGAT
CGCGATGGGAAAAAATTCGGTTAAGGCCAGGGGGAAAGAAAAAATATAAATTAAAACATATAGTATGG
GCAAGCAGGGAGCTAGAACGATTCGCAGTTAATCCTGGCCTGTTAGAAACATCAGAAGGCTGTAGACA
AATACTGGGACAGCTACAACCATCCCTTCAGACAGGATCAGAAGAACTTAGATCATTATATAATACAGT
AGCAACCCTCTATTGTGTGCATCAAAGGATAGAGATAAAAGACACCAAGGAAGCTTTAGACAAGATAG
AGGAAGAGCAAAACAAAAGTAAGACCACCGCACAGCAAGCGGCCGCTGATCTTCAGACCTGGAGGA
GGAGATATGAGGGACAATTGGAGAAGTGAATTATATAAATATAAAGTAGTAAAAATTGAACCATTAGGA
GTAGCACCCACCAAGGCAAAGAGAAGAGTGGTGCAGAGAGAAAAAAGAGCAGTGGGAATAGGAGCT
TTGTTCCTTGGGTTCTTGGGAGCAGCAGGAAGCACTATGGGCGCAGCGTCAATGACGCTGACGGTACA
GGCCAGACAATTATTGTCTGGTATAGTGCAGCAGCAGAACAATTTGCTGAGGGCTATTGAGGCGCAAC
AGCATCTGTTGCAACTCACAGTCTGGGGCATCAAGCAGCTCCAGGCAAGAATCCTGGCTGTGGAAAGA
TACCTAAAGGATCAACAGCTCCTGGGGATTTGGGGTTGCTCTGGAAAACTCATTTGCACCACTGCTGTG
CCTTGGAATGCTAGTTGGAGTAATAAATCTCTGGAACAGATTTGGAATCACACGACCTGGATGGAGTGG
GACAGAGAAATTAACAATTACACAAGCTTAATACACTCCTTAATTGAAGAATCGCAAAACCAGCAAGA
AAAGAATGAACAAGAATTATTGGAATTAGATAAATGGGCAAGTTTGTGGAATTGGTTTAACATAACAAA
TTGGCTGTGGTATATAAAATTATTCATAATGATAGTAGGAGGCTTGGTAGGTTTAAGAATAGTTTTTGCTG
TACTTTCTATAGTGAATAGAGTTAGGCAGGGATATTCACCATTATCGTTTCAGACCCACCTCCCAACCCC
GAGGGGACCCGACAGGCCCGAAGGAATAGAAGAAGAAGGTGGAGAGAGAGACAGAGACAGATCCAT
TCGATTAGTGAACGGATCTCGACGGTATCGGTTAACTTTTAAAAGAAAAGGGGGGATTGGGGGGTACA
GTGCAGGGGAAAGAATAGTAGACATAATAGCAACAGACATACAAACTAAAGAATTACAAAAACAAATT

ACAAAATTCAAAATTTTATCGATGCCTCCCCGTCACCACCCCCCCCAACCCGCCCCGACCGGAGCTGA

GAGTAATTCATACAAAAGGACTCGCCCCTGCCTTGGGGAATCCCAGGGACCGTCGTTAAACTCCCACT

AACGTAGAACCCAGAGATCGCTGCGTTCCCGCCCCCTCACCCGCCCGCTCTCGTCATCACTGAGGTGG

AGAAGAGCATGCGTGAGGCTCCGGTGCCCGTCAGTGGGCAGAGCGCACATCGCCCACAGTCCCCGAG

AAGTTGGGGGGAGGGGTCGGCAATTGAACCGGTGCCTAGAGAAGGTGGCGCGGGGTAAACTGGGAA

AGTGATGTCGTGTACTGGCTCCGCCTTTTTCCCGAGGGTGGGGGAGAACCGTATATAAGTGCAGTAGTC

GCCGTGAACGTTCTTTTTCGCAACGGGTTTGCCGCCAGAACACAGGTAAGTGCCGTGTGTGGTTCCCG

CGGGCCTGGCCTCTTTACGGGTTATGGCCCTTGCGTGCCTTGAATTACTTCCACGCCCCTGGCTGCAGT

ACGTGATTCTTGATCCCGAGCTTCGGGTTGGAAGTGGGTGGGAGAGTTCGAGGCCTTGCGCTTAAGGA

GCCCCTTCGCCTCGTGCTTGAGTTGAGGCCTGGCCTGGGCGCTGGGGCCGCCGCGTGCGAATCTGGTG

GCACCTTCGCGCCTGTCTCGCTGCTTTCGATAAGTCTCTAGCCATTTAAAATTTTTGATGATATCCTGCG

ACGCTTTTTTTCTGGCAAGATAGTCTTGTAAATGCGGGCCAAGATCTGCACACTGGTATTTCGGTTTTTG

GGGCCGCGGGCGGCGACGGGGCCCGTGCGTCCCAGCGCACATGTTCGGCGAGGCGGGGCCTGCGAGC

GCGGCCACCGAGAATCGGACGGGGGTAGTCTCAAGCTGGCCGGCCTGCTCTGGTGCCTGGCCTCGCG

CCGCCGTGTATCGCCCCGCCCTGGGCGGCAAGGCTGGCCCGGTCGGCACCAGTTGCGTGAGCGGAAA

GATGGCCGCTTCCCGGCCCTGCTGCAGGGAGCTCAAAATGGAGGACGCGGCGCTCGGGAGAGCGGGC

GGGTGAGTCACCCACACAAAGGAAAAGGGCCTTTCCGTCCTCAGCCGTCGCTTCATGTGACTCCACGG

AGTACCGGGCGCCGTCCAGGCACCTCGATTAGTTCTCGAGCTTTTGGAGTACGTCGTCTTTAGGTTGGG

GGGAGGGGTTTTATGCGATGGAGTTTCCCCACACTGAGTGGGTGGAGACTGAAGTTAGGCCAGCTTGG

CACTTGATGTAATTCTCCTTGGAATTTGCCCTTTTTGAGTTTGGATCTTGGTTCATTCTCAAGCCTCAGA

CAGTGGTTCAAAGTTTTTTTCTTCCATTTCAGGTGTCGTGAAAACTACCCCTGAGCTCCTTAAGGTTAA

CTCTAGAGAATTCGGATCCAATATTCCCGGGCTCGAGCCGAAGGCCACCATGGAAACCGACACCCTGC

TGCTGTGGGTGCTGCTCCTGTGGGTCCCCGGCTCTACCGGCGACGGCGGCAGCGACTACAAGGACGAC

GATGATAAGGGCGGCTCCGGCGGAAGCacgcgtGCCAGCAAGCTGTACAGCGGCAGCGGCGGCTCTGGA

GGCAGCGGCGGATCTGCTGTGGGCCAGGATACACAGGAGGTGATCGTGGTGCCACACAGCCTGCCTTT

CAAGGTGGTGGTTATCAGCGCCATCCTGGCCCTGGTGGTGCTGACCATCATTAGCCTGATCATCCTGATC

ATGCTGTGGCAGAAAAAGCCTCGGTGACTGACTGAACCGGTGGTACCGATCCGGCGCGCCCTCCGGCC

TAGGGATAACAGGGTAATCCGCTAGCCCCTCTCCCTCCCCCCCCCCTAACGTTACTGGCCGAAGCCGCT

TGGAATAAGGCCGGTGTGCGTTTGTCTATATGTTATTTTCCACCATATTGCCGTCTTTTGGCAATGTGAG

GGCCCGGAAACCTGGCCCTGTCTTCTTGACGAGCATTCCTAGGGGTCTTTCCCCTCTCGCCAAAGGAAT
GCAAGGTCTGTTGAATGTCGTGAAGGAAGCAGTTCCTCTGGAAGCTTCTTGAAGACAAACAACGTCTG
TAGCGACCCTTTGCAGGCAGCGGAACCCCCCACCTGGCGACAGGTGCCTCTGCGGCCAAAAGCCACG
TGTATAAGATACACCTGCAAAGGCGGCACAACCCCAGTGCCACGTTGTGAGTTGGATAGTTGTGGAAA
GAGTCAAATGGCTCTCCTCAAGCGTATTCAACAAGGGGCTGAAGGATGCCCAGAAGGTACCCCATTGT
ATGGGATCTGATCTGGGGCCTCGGTACACATGCTTTACATGTGTTTAGTCGAGGTTAAAAAAACGTCTA
GGCCCCCCGAACCACGGGGACGTGGTTTTCCTTTGAAAAACACGATGATAATATGGCCACACTAGAGA
TCCACCGGTCGCCACCATGACCGAGTACAAGCCCACGGTGCGCCTCGCCACCCGCGACGACGTCCCCA
GGGCCGTACGCACCCTCGCCGCCGCGTTCGCCGACTACCCCGCCACGCGCCACACCGTCGATCCGGAC
CGCCACATCGAGCGGGTCACCGAGCTGCAAGAACTCTTCCTCACGCGCGTCGGGCTCGACATCGGCAA
GGTGTGGGTCGCGGACGACGGCGCCGCGGTGGCGGTCTGGACCACGCCGGAGAGCGTCGAAGCGGG
GGCGGTGTTCGCCGAGATCGGCCCGCGCATGGCCGAGTTGAGCGGTTCCCGGCTGGCCGCGCAGCAA
CAGATGGAAGGCCTCCTGGCGCCGCACCGGCCCAAGGAGCCCGCGTGGTTCCTGGCCACCGTCGGCG
TCTCGCCCGACCACCAGGGCAAGGGTCTGGGCAGCGCCGTCGTGCTCCCCGGAGTGGAGGCGGCCGA
GCGCGCCGGGGTGCCCGCCTTCCTGGAGACCTCCGCGCCCCGCAACCTCCCCTTCTACGAGCGGCTCG
GCTTCACCGTCACCGCCGACGTCGAGGTGCCCGAAGGACCGCGCACCTGGTGCATGACCCGCAAGCC
CGGTGCCTGAGCGGCCGCGTCGACAATCAACCTCTGGATTACAAAATTTGTGAAAGATTGACTGGTATT
CTTAACTATGTTGCTCCTTTTACGCTATGTGGATACGCTGCTTTAATGCCTTTGTATCATGCTATTGCTTCC
CGTATGGCTTTCATTTTCTCCTCCTTGTATAAATCCTGGTTGCTGTCTCTTTATGAGGAGTTGTGGCCCGT
TGTCAGGCAACGTGGCGTGGTGTGCACTGTGTTTGCTGACGCAACCCCCACTGGTTGGGGCATTGCCA
CCACCTGTCAGCTCCTTTCCGGGACTTTCGCTTTCCCCCTCCCTATTGCCACGGCGGAACTCATCGCCG
CCTGCCTTGCCCGCTGCTGGACAGGGGCTCGGCTGTTGGGCACTGACAATTCCGTGGTGTTGTCGGGG
AAGCTGACGTCCTTTCCATGGCTGCTCGCCTGTGTTGCCACCTGGATTCTGCGCGGGACGTCCTTCTGC
TACGTCCCTTCGGCCCTCAATCCAGCGGACCTTCCTTCCCGCGGCCTGCTGCCGGCTCTGCGGCCTCTT
CCGCGTCTTCGCCTTCGCCCTCAGACGAGTCGGATCTCCCTTTGGGCCGCCTCCCCGCCTGGAATTCGA
GCTCGGTACCTTTAAGACCAATGACTTACAAGGCAGCTGTAGATCTTAGCCACTTTTTAAAAGAAAAGG
GGGGACTGGAAGGGCTAATTCACTCCCAACGAAGACAAGATCTGCTTTTTGCTTGTACTGGGTCTCTCT
GGTTAGACCAGATCTGAGCCTGGGAGCTCTCTGGCTAACTAGGGAACCCACTGCTTAAGCCTCAATAA
AGCTTGCCTTGAGTGCTTCAAGTAGTGTGTGCCCGTCTGTTGTGTGACTCTGGTAACTAGAGATCCCTC

AGACCCTTTTAGTCAGTGTGGAAAATCTCTAGCAGTAGTAGTTCATGTCATCTTATTATTCAGTATTTATA

ACTTGCAAAGAAATGAATATCAGAGAGTGAGAGGAACTTGTTTATTGCAGCTTATAATGGTTACAAATA

AAGCAATAGCATCACAAATTTCACAAATAAAGCATTTTTTTCACTGCATTCTAGTTGTGGTTTGTCCAAA

CTCATCAATGTATCTTATCATGTCTGGCTCTAGCTATCCCGCCCCTAACTCCGCCCATCCCGCCCCTAACT

CCGCCCAGTTCCGCCCATTCTCCGCCCCATGGCTGACTAATTTTTTTTATTTATGCAGAGGCCGAGGCCG

CCTCGGCCTCTGAGCTATTCCAGAAGTAGTGAGGAGGCTTTTTTGGAGGCCTAGGGACGTACCCAATTC

GCCCTATAGTGAGTCGTATTACGCGCGCTCACTGGCCGTCGTTTTACAACGTCGTGACTGGGAAAACCC

TGGCGTTACCCAACTTAATCGCCTTGCAGCACATCCCCCTTTCGCCAGCTGGCGTAATAGCGAAGAGGC

CCGCACCGATCGCCCTTCCCAACAGTTGCGCAGCCTGAATGGCGAATGGGACGCGCCCTGTAGCGGCG

CATTAAGCGCGGCGGGTGTGGTGGTTACGCGCAGCGTGACCGCTACACTTGCCAGCGCCCTAGCGCCC

GCTCCTTTCGCTTTCTTCCCTTCCTTTCTCGCCACGTTCGCCGGCTTTCCCCGTCAAGCTCTAAATCGGG

GGCTCCCTTTAGGGTTCCGATTTAGTGCTTTACGGCACCTCGACCCCAAAAAACTTGATTAGGGTGATG

GTTCACGTAGTGGGCCATCGCCCTGATAGACGGTTTTTCGCCCTTTGACGTTGGAGTCCACGTTCTTTA

ATAGTGGACTCTTGTTCCAAACTGGAACAACACTCAACCCTATCTCGGTCTATTCTTTTGATTTATAAGG

GATTTTGCCGATTTCGGCCTATTGGTTAAAAAATGAGCTGATTTAACAAAAATTTAACGCGAATTTTAAC

AAAATATTAACGCTTACAATTTAGGTGGCACTTTTCGGGGAAATGTGCGCGGAACCCCTATTTGTTTATT

TTTCTAAATACATTCAAATATGTATCCGCTCATGAGACAATAACCCTGATAAATGCTTCAATAATATTGAA

AAAGGAAGAGTATGAGTATTCAACATTTCCGTGTCGCCCTTATTCCCTTTTTTGCGGCATTTTGCCTTCC

TGTTTTTGCTCACCCAGAAACGCTGGTGAAAGTAAAAGATGCTGAAGATCAGTTGGGTGCACGAGTGG

GTTACATCGAACTGGATCTCAACAGCGGTAAGATCCTTGAGAGTTTTCGCCCCGAAGAACGTTTTCCAA

TGATGAGCACTTTTAAAGTTCTGCTATGTGGCGCGGTATTATCCCGTATTGACGCCGGGCAAGAGCAAC

TCGGTCGCCGCATACACTATTCTCAGAATGACTTGGTTGAGTACTCACCAGTCACAGAAAAGCATCTTA

CGGATGGCATGACAGTAAGAGAATTATGCAGTGCTGCCATAACCATGAGTGATAACACTGCGGCCAACT

TACTTCTGACAACGATCGGAGGACCGAAGGAGCTAACCGCTTTTTTGCACAACATGGGGGATCATGTA

ACTCGCCTTGATCGTTGGGAACCGGAGCTGAATGAAGCCATACCAAACGACGAGCGTGACACCACGAT

GCCTGTAGCAATGGCAACAACGTTGCGCAAACTATTAACTGGCGAACTACTTACTCTAGCTTCCCGGCA

ACAATTAATAGACTGGATGGAGGCGGATAAAGTTGCAGGACCACTTCTGCGCTCGGCCCTTCCGGCTG

GCTGGTTTATTGCTGATAAATCTGGAGCCGGTGAGCGTGGGTCTCGCGGTATCATTGCAGCACTGGGGC

CAGATGGTAAGCCCTCCCGTATCGTAGTTATCTACACGACGGGGAGTCAGGCAACTATGGATGAACGAA

ATAGACAGATCGCTGAGATAGGTGCCTCACTGATTAAGCATTGGTAACTGTCAGACCAAGTTTACTCAT
ATATACTTTAGATTGATTTAAAACTTCATTTTTAATTTAAAAGGATCTAGGTGAAGATCCTTTTTGATAAT
CTCATGACCAAAATCCCTTAACGTGAGTTTTCGTTCCACTGAGCGTCAGACCCCGTAGAAAAGATCAA
AGGATCTTCTTGAGATCCTTTTTTTCTGCGCGTAATCTGCTGCTTGCAAACAAAAAAACCACCGCTACC
AGCGGTGGTTTGTTTGCCGGATCAAGAGCTACCAACTCTTTTTCCGAAGGTAACTGGCTTCAGCAGAG
CGCAGATACCAAATACTGTTCTTCTAGTGTAGCCGTAGTTAGGCCACCACTTCAAGAACTCTGTAGCAC
CGCCTACATACCTCGCTCTGCTAATCCTGTTACCAGTGGCTGCTGCCAGTGGCGATAAGTCGTGTCTTAC
CGGGTTGGACTCAAGACGATAGTTACCGGATAAGGCGCAGCGGTCGGGCTGAACGGGGGGTTCGTGC
ACACAGCCCAGCTTGGAGCGAACGACCTACACCGAACTGAGATACCTACAGCGTGAGCTATGAGAAA
GCGCCACGCTTCCCGAAGGGAGAAAGGCGGACAGGTATCCGGTAAGCGGCAGGGTCGGAACAGGAG
AGCGCACGAGGGAGCTTCCAGGGGGAAACGCCTGGTATCTTTATAGTCCTGTCGGGTTTCGCCACCTC
TGACTTGAGCGTCGATTTTTGTGATGCTCGTCAGGGGGGCGGAGCCTATGGAAAAACGCCAGCAACGC
GGCCTTTTTACGGTTCCTGGCCTTTTGCTGGCCTTTTGCTCACATGTTCTTTCCTGCGTTATCCCCTGATT
CTGTGGATAACCGTATTACCGCCTTTGAGTGAGCTGATACCGCTCGCCGCAGCCGAACGACCGAGCGC
AGCGAGTCAGTGAGCGAGGAAGCGGAAGAGCGCCCAATACGCAAACCGCCTCTCCCCGCGCGTTGGC
CGATTCATTAATGCAGCTGGCACGACAGGTTTCCCGACTGGAAAGCGGGCAGTGAGCGCAACGCAATT
AATGTGAGTTAGCTCACTCATTAGGCACCCCAGGCTTTACACTTTATGCTTCCGGCTCGTATGTTGTGTG
GAATTGTGAGCGGATAACAATTTCACACAGGAAACAGCTATGACCATGATTACGCCAAGCGCGCAATTA
ACCCTCACTAAAGGGAACAAAAGCTGGAGCTGC

Construction of cell-displayed DRP lentivirus plasmid library

The procedures of constructing cell-displayed DRP library-1 (CPPCX5CX5CX5CPPC; X is any amino acid encoded by NNK) as a typical example were described below. Firstly, a chemosynthetic 110 bp oligonucleotide was co-incubated with the reverse primer in 95 °C for 5 min followed by cooling in ice to achieve annealing. After that, it formed a 110 bp double-stranded DNA fragment through extension (4 h, 37 °C; 15 min, 65 °C) with Klenow fragment. The double-stranded DNA fragment was extracted by DNA Extraction Solution. Next, the double-stranded DNA fragment and cell-displayed lentiviral vector were digested with *MluI* (7 h, 37 °C) and *Bsp1407I* (7 h, 37 °C). Subsequently, the digested double-stranded DNA fragment and cell-displayed lentiviral vector were purified by polyacrylamide gel electrophoresis (PAGE) and agarose gel electrophoresis respectively. Afterward, the *MluI*/*Bsp1407I*-digested DNA fragment was ligated to the *MluI*/*Bsp1407I*-digested cell-displayed lentiviral vector

(ratio of insert and vector: 2/1) in a ligation system containing T4 enzyme overnight at 16 °C. The ligated product was purified then transformed into electrocompetent *E. coli* Stabl3 cells by electroporation (200 Ω, 2.5 kV). The transformed cells were plated on 2×YT/Ampicillin agar plates and incubated at 37 °C for 16 h. The library capacity was evaluated by counting the clone number of gradient diluted 2×YT/Ampicillin agar plates (library size: 3.0×10^6 pfu). ~10 clones were randomly selected for Sanger sequencing to evaluate the accuracy of cell-displayed DRP lentiviral plasmid library.

Preparation of the cell-displayed DRP lentiviral library

Typically, HEK293T cells were of 70-80% confluent at the time of transfection. Cells were transiently transfected with a DNA mixture of cell-displayed DRP lentivirus plasmid library and packaging plasmid pMDLg, pRSV-REWV, envelop plasmid pMD2.G-VSV at a ratio of 3:1:1:1 using the Lipofectamine 2000 reagent according to the recommendations of the manufacturer. The medium was changed the next day and lentivirus supernatants was collected twice at 48 h and 72 h post-transfection. The supernatants were centrifuged at 2000 rpm for 20 min at 4 °C to remove cell debris and filtered through 0.45 μmPES filters. After that, a lentivirus concentration reagent (20% v/v of supernatants) was added to lentivirus supernatants to concentrate lentivirus, then mixed and placed at 4 °C refrigerator overnight for sedimentation. The next day, the solution was centrifuged at 6000 *g* for 30 min at 4 °C and the supernatant was removed. Finally, lentivirus pellets were resuspended with 10% FBS DMEM medium, then aliquoted 100 μL per tube and feezed at -80 °C for use.

Determination of the cell-displayed DRP lentiviral library titer

The titer of the cell-displayed DRP lentiviral library-1 was determined by Quantitative PCR (qPCR)-Based Assay according to the literature procedure[1, 2]. Two pairs of primers (primer 3, 4, 5 and 6) which can respectively amplify a lentiviral-specific transgene (WPRE gene) and a single copy gene-specific reference gene (36B4 gene) were designed for Quantitative PCR-Based Assay. A standard plasmid pUC57 WPRE&36B4 containing WPRE gene and 36B4 gene was designed and purchased from Genscript (Nanjing, China). Typically, HEK293T cells were plated at a density of 6×10^5 cells per well in a 6-well plate before transduction. The next day, the number of cells in one of the 6-well in the plate was counted by using a hemacytometer. And then the medium was removed from other wells and replaced with 1 mL of fresh medium containing 10 μg/mL of polybrene. Cells were transduced by adding 0.2, 2 and 20 μL aliquots of a concentrated cell-displayed DRP lentiviral library-1 stock per well, respectively. After that, the 6-well plate was tilted to distribute the lentivirus and returned to the cell culture incubator. The supernatant of the plate was removed and then replaced 0.5 mL of medium containing 10 U/mL DNaseI (incubate for 15 min at 37 °C) after 20 h

of transduction. The medium of the plate was removed and then replaced with 1 ml of fresh medium and continue to incubate cells for another 48 h. Cells was digested by using 0.5 mL of a 0.25% trypsin-EDTA solution for 1 min at 37 °C and then collected. To isolate high-molecular weight genomic DNA (gDNA) from transduced HEK293T cells using SteadyPure Universal Genomic DNA Extraction Kit according to the manufacturer's instructions. The preparation of all qPCR reactions was followed the instruction of SYBR® Green Premix Pro Taq HS qPCR Kit. The qPCR-based assay was performed with an CFX96 Touch Real-Time PCR Detection System (Set cycling conditions as follows: 30 s at 95 °C, 40 cycles of 95 °C for 5 s and 60 °C for 30 s, then dissociation stage). Finally, the Ct value was obtained after the qPCR reaction, and the titer of cell-displayed DRP lentiviral library-1 was calculated according to the following formula after data analysis. Calculate titers (integration units per ml, TU/mL) according to the following formula:

$$\text{Titers (TU/mL)} = (C\times N\times D\times 1000)/V$$

Where C=2×WPRE gene copies/36B4 gene copies (lentiviral copies per genome), N=number of cells at time of transduction, D=dilution of lentivirus library preparation, V=volume of diluted lentivirus library added in each well for transduction.

Considering that the cell-displayed DRP lentiviral library-2 containing a copGFP reporter gene, a relatively simple and accurate GFP fluorescence counting method (combined with flow cytometry) was used by Flow cytometric (FACS) method according to the literature procedure[1].

Protein quality control-based selection of the cell-displayed DRP lentiviral library-1

HEK293T cells were plated at a density of 6×10^6 cells per T75 Flasks so that the cells were 50-60% confluent on the next day (Prepare two T75 Flasks). And then the medium was removed from two T75 Flasks and replaced with 16 mL of fresh DMEM medium containing 10 μg/mL of polybrene and 1.9×10^7 TU cell-displayed DRP lentiviral library-1 (The amount of virus used for MOI<0.7). ~68 h post-transduction, cells were washed with 1×PBS and treated with 3 mL Non-enzyme cell detached solution for 10 min at RT. The treated cells were washed twice and resuspended with 12 mL 1×PBS containing 5% BSA for 30 min at RT using a slowly rotating wheel. After that, cells were pelleted and resuspended with 6 mL 1×PBS with 3% BSA containing a primary antibody (Monoclonal ANTI-FLAG® M2 antibody, 1:500 [v/v] dilution) for 1.5 h at RT using a slowly rotating wheel. Cells were then washed three times (6 mL 1×PBS with 3% BSA, 5 min per wash). All subsequent washing steps used this procedure. Subsequently, cells were incubated with 6 mL 1×PBS with 3% BSA containing a secondary antibody (CoraLite488–conjugated Affinipure Goat Anti-Mouse IgG(H+L), 1:500 [v/v] dilution) for 1 h at RT using a slowly rotating wheel and then

washed. A total of ~8×10^7 stained cells were subjected to separation using a BD FACS Aria III flow sorter. For the first-round selection, ~15.5% positive cells (cells with high fluorescence intensity) of total cells were collected and then further culture with 15% FBS DMEM with the addition of Penicillin and Streptomycin for the next round selection. Four iterative rounds of selection were carried out.

Protein quality control-based selection of the cell-displayed DRP lentiviral library-2

1.65×10^7 TU cell-displayed DRP lentiviral library-2 at MOI<0.7 was added to the pre-prepared HEK293T cells (~4.06×10^7) in 24 mL of fresh DMEM medium containing 10 μg/mL of polybrene. ~68 h post-transduction, cells were washed with 1×PBS and treated with 3 mL Non-enzyme cell detached solution for 10 min at RT. The treated cells were washed twice and resuspended with 12 mL 1×PBS containing 5% BSA for 30 min at RT using a slowly rotating wheel. After that, cells were pelleted and resuspended with 6 mL 1×PBS with 3% BSA containing a primary antibody (Monoclonal ANTI-FLAG® M2 antibody, 1:500 [v/v] dilution) for 1.5 h at RT using a slowly rotating wheel. Cells were then washed. Subsequently, cells were incubated with 6 mL 1×PBS with 3% BSA containing a secondary antibody (Goat Anti-Mouse IgG H&L (Alexa Fluor® 647), 1:2000 [v/v] dilution) for 1 h at RT using a slowly rotating wheel and then washed. A total of ~1×10^8 stained cells were subjected to separation using a BD FACS Aria III flow sorter. For the first-round selection, ~7.67% positive cells (the populations with lower GFP and higher surface FLAG-stain fluorescence were sorted) of total cells were collected and then further culture with 15% FBS DMEM with the addition of Penicillin and Streptomycin for the next round selection. Four iterative rounds of selection were carried out.

Next-generation sequencing (NGS) and bioinformatics analysis

The genomic DNA (gDNA) was extracted from transduced HEK293T cell population using SteadyPure Universal Genomic DNA Extraction Kit according to the manufacturer's instructions. The genes encoded peptides from gDNA after each round were amplified by using primers with different barcodes. A typical PCR reaction in a volume of 100 μL contained final concentrations of 0.2 μM of primer, 1×PrimeSTAR HS DNA polymerase buffer, 250 μM dNTPs, 1~10 ng/μL gDNA template and 2 Unit PrimeSTAR HS DNA polymerase. The following program was used: initial denaturation for 3 min at 95°C, 29 cycles of 30 s at 95°C, 30 s at 61°C, 30 s at 72°C, and final elongation for 10 min at 72°C. PCR products were cut out and purified from a 1.5% agarose gel using a commercial agarose gel purification kit (E.Z.N.A® Gel Extraction Kit, OMEGA BIO-TEK, USA). Sequencing was performed by Novogene Co., Ltd. on an Novaseq 6000 sequencing platform using PE250 sequencing strategy. The NGS data were processed and analyzed using MatLab scripts (the translation script of "AmberE=setfiled (GeneticCode, 'TAG', '*')" has been changed prior to

processing the data) according to the literature procedure[3].

Flow cytometry analysis of cell display efficiency of peptides selected from P4 cell population

The genomic DNA (gDNA) was extracted from P4 cell population (After R4 Sorts) using SteadyPure Universal Genomic DNA Extraction Kit according to the manufacturer's instructions. The DNA sequence of peptides were amplified with primers 7 and 8 using gDNA (P4 cell population) as a template and introduced by Gibson assembly into *NheI* and *BamHI* digested pLV SP-flag-PDFGRβ TM fragment. The Gibson assembly product was electroporated into electrocompetent *E. coli* Stabl3 cells by using the bacteria (*E. coli*) mode of the electroporator (Bio-Rad MicroPulser). Subsequently, the cells were recovered for 2 h and plated on large 2×YT/Ampicillin agar plates and incubated at 37 °C overnight. ~30 clones were randomly picked up for sequencing and then 12 peptides were randomly selected for the following cell display efficiency validation. Firstly, the 12 plasmids of clones corresponding to 12 peptides were isolated respectively by QIAprep spin Miniprep Kit. After that, 12 cell-displayed lentiviruses corresponding to 12 peptides were packaged and transduced into HEK293T cells in a manner of MOI<1. After ~68 h of incubation, transduced cells were detached using non-enzyme cell detached solution. Cells were then pelleted by centrifuge (900 rpm, 6 min) and resuspended in 1 mL PBS with 5% BSA, and incubated at RT for 30 min using a slowly rotating wheel. After incubation, cells were then pelleted by centrifuge (900 rpm, 6 min) and resuspended in 1 mL PBS with 3% BSA containing Monoclonal ANTI-FLAG® M2 antibody produced in mouse (1:500 [v/v] dilution). Cells were then incubated at RT for 1.5 h using a slowly rotating wheel and then washed three times (1 mL PBS with 3% BSA, 5 min per wash). Subsequently, cells were stained in 1 mL PBS with 3% BSA containing CoraLite488–conjugated Affinipure Goat Anti-Mouse IgG(H+L) (1:500 [v/v] dilution). Cells were then incubated at RT for 1 h using a slowly rotating wheel and then washed three times (1 mL PBS with 3% BSA, 5 min per wash). Finally, all cell samples were pelleted by centrifuge (900 rpm, 6 min) and then resuspended in 500 μL DMEM prior to analysing by flow cytometry (BD FACS Aria III).

Construction of the Error-Prone PCR phage-displayed peptide library

Phage-displayed peptide libraries was constructed as described previously. Firstly, Primer 9 and 10 were used to amplify the encoded peptides DNA fragment (181 bp) with the genomic DNA which extracted from cell population (P9) as a template. After that, in order to obtain a phage-displayed peptide library with a higher mutation capacity, eight rounds sequential Error-Prone PCR were performed with primer 9 and 10 by using the PCR product of encoded peptides DNA fragment as a starting template (a Error-Prone PCR reaction in a volume of 30 μL contains 1 μL 10 μM of primer 9, 1 μL 10 μM of primer 10, 3 μL 10×Taq DNA polymerase buffer with $MgCl_2$, 0.6 μL 10 mM dNTPs,

6 μL 250 μM $MnCl_2$, 4 μL 10 mM dGTP, 1 ng template and 3 μL low fidelity Taq DNA polymerase). The final round of Error-Prone PCR product was further amplified with primer 9 and 10 by using a high fidelity PrimeSTAR® HS DNA Polymerase. The amplified Error-Prone PCR product (~10 μg) was then digested by using *SfiI* and *NotI* to generate a 154 bp insert fragment (~2 μg). The insert fragment was cloned into the *SfiI*/*NotI*-digested phage displayed vector (pCantab 5e, ~10 μg) by using T4 ligation enzyme. Subsequently, the ligated DNA (~7 μg) was purified and then electroporated into electrocompetent *E. coli* TG1 cells by using the bacteria (*E. coli*) mode of the electroporator (Bio-Rad MicroPulser). The capacity of the library was determined by measuring the total volume of electroporated cells, taking an aliquot of 20 μL and plating a series of 10-fold dilutions on small 2×YT/Ampicillin agar plates. The rest of the cells were plated on large 2×YT/ Ampicillin agar plates and incubated at 37 °C overnight. Finally, the library capacity was evaluated by counting the clone number of gradient diluted 2×YT/Ampicillin agar plates (library size: 2.0×10^9 pfu). The *E. coli* TG1 cells were scraped off the plates in 20 mL of 2×YT/Ampicillin medium containing 20% glycerol. Aliquots of 1 mL were stored at −80 °C as glycerol stocks.

Biotinylation of Keap1 kelch domain protein

Keap1 kelch domain protein (Residues 321-609, Homo Sapiens) was biotinylated at a concentration of 2.67 mg/mL which reacted with a 5-fold molar excess of EZ-Link™ Sulfo-NHS-LC-Biotin in 10 mM PBS buffer (pH 7.4) at room temperature for 30 min. Excess of biotin was removed on a desalting column using a FPLC system.

Phage panning

Phage peptide library were produced and purified as previously described[4]. A typical selection experiment was described below. Phage peptide library was produced in a 200 mL 2×YT medium. Firstly, 50 μL magnetic streptavidin beads (or neutravidin-coated magnetic beads for the second-round panning) were washed three times with 500 μL Binding buffer (10 mM Tris-HCl, 150 mM NaCl, 10 mM $MgCl_2$, 1 mM $CaCl_2$, pH 7.4) with the help of a magnetic. Magnetic streptavidin beads were resuspended in 50 μL Binding buffer and then divided into two aliquots (25 μL each) in a 1.5 mL low-adsorption centrifugal tubes. Biotinylated Keap1 kelch domain (5 μg, 25 μL) was immobilized on one of the magnetic streptavidin beads aliquots and the same volume of 1×PBS (without target protein) was added to another beads aliquots by incubation for 15 min at room temperature. After that, two tubes of beads were washed three times with 500 μL Binding buffer and then blocked in 300 μL Binding buffer and 150 μL of Blocking buffer (Binding buffer with 0.3% v/v Tween-20 and 3% w/v BSA), then incubated on a slowly rotating wheel at room temperature for 2 hours. In parallel, phage peptide library (>10^{12}~10^{13} TU) was blocked in 4.5 mL of the same buffer and incubated on a slowly rotating wheel at room temperature for 2 hours. Subsequently, the beads coated with or

without target protein were then added to half of the blocked phage respectively and incubated for 30 min on a rotating wheel. The beads were washed three times (Round 2, washed six times; Round 3, washed nine times) with Washing buffer (Binding buffer with 0.1% v/v Tween-20) and twice with Binding buffer. After that, phage bound to the beads were eluted by adding 200 μL Elution buffer (50 mM Glycine, pH 2.2) and incubating for 5 min. Eluted phage was neutralized by addition of 20 μL Neutralization buffer (1 M Tris-Cl, pH 8.0). 20 μL of the eluted phage from coated target protein beads was used to infect exponentially growing TG1 *E. coli* for monitoring the phage titer, the remaining of eluted phage was then incubated with ~25 mL of exponentially growing TG1 *E. coli* cells (OD_{600}=0.4) for 90 min. Eluted phage infected TG1 *E. coli* cells were plated on 2YT/Ampicillin agar plate and incubated at 37 °C. The cells were harvested the next day with 2YT media which used to produce phage to next round of panning.

Synthesis of peptides

All peptides were prepared using Fmoc-solid phase peptide synthesis on a CEM Liberty Blue automated microwave peptide synthesizer. Rink-amide MBHA resin at a scale of 0.025 mmol was used for the synthesis. A typical synthesis cycle consists of (a) Fmoc-deprotection (20% piperidine in DMF), (b) DMF washings, (c) coupling (5 eq. Fmoc−Xaa−OH, 5 eq. DIC and 5 eq. Oxyma Pure in DMF), and (d) DMF washings. Then peptides were cleaved from the resin and deprotected by treating with a 4 mL cleavage cocktail (TFA/thioanisole/EDT/H_2O/Phenol= 87.5:5:2.5:2.5:2.5) for shaking 4 h at 37 °C. After that, the peptides were precipitated with cold diethyl ether, collected by centrifugation (The precipitated peptides should be washed with cold diethyl ether for three times), then purified using a semi-preparative reverse-phase HPLC system. Peptides (>95% purity) were identified by a Bruker autoflex maX MALDI-TOF mass spectroscopy.

Oxidative folding of peptides

The fully reduced peptides were oxidized in various buffers. Reduced peptides (**drp1 and 4**, 10 μM) was dissolved in 100 mM phosphate buffer (pH 7.4) containing 0.2 mM GSSG. Reduced peptides (**drp2 and 3**, **k-1**, **k-2**, 10 μM) was dissolved in 100 mM phosphate buffer (pH 7.4) containing 6 M Gu·HCl and 0.2 mM GSSG. The reaction mixture was placed on a shaker overnight at 37 °C. After that, oxidative folding of all the peptides was further analysed using HPLC and mass spectrometry.

NMR experiment of drp1

Drp1 were dissolved in 80% perdeuterated acetonitrile and 20% H_2O at a final concentration of about 1 mM. NMR experiments were carried out at 302 K on Bruker AVANCE III 850 MHz equipped with a cryogenic triple-resonance probe. Two dimensional (2D) 1H, 15N/13C HSQC spectra were recorded to obtain chemical shifts of heavy atoms

in backbone and side-chains. 2D 1H-1H TOCSY (mixing time, 80 ms) and 2D 1H-1H COSY spectra were acquired to the sequential assignment of peptides, and 2D 1H-1H NOESY spectra with a mixing time of 300 ms were carried out for structure calculation. The NMR data were processed using NMRPipe/NMRDraw and analyzed using NMRFAM-SPARKY[5]. More than 95% of all NOE cross-peaks were assigned manually. The backbone dihedral angle restraints were obtained using programs TALOS-N based on chemical shifts of backbone resonances[6]. The distance constraints were deduced from volume integration of NOE crosspeaks and carried out with ARIA2.3.2 and CNS1.21[7, 8]. All of the ensemble of 15 lowest-energy structures were generated from a total of 150 structures in last iteration for each run. The structures were visualized in PyMol, and the quality of structures was analyzed using PROCHECK[9].

Surface plasmon resonance (SPR) assays

The binding affinity of peptides targeting Keap1 were characterized by Surface plasmon resonance (SPR) analysis using a Biacore T200 (GE healthcare) at 25°C. In this assay, the running buffer was 10 mM phosphate buffer (pH = 7.4) with 50 μM EDTA, 0.05% Tween 20. Biotinylated Keap1 was immobilized on the surface of a Biacore CAP Chip using a Biotin CAPture Kit (GE healthcare). Serially diluted samples (50, 100, 200, 400, 800, 1600, 3200 nM of oxidized peptide) for measuring at a flow rate of 30 μL/min, and binding affinity and kinetics constants were quantified by a multiple cycle kinetics method. Kinetics data were analyzed using a 1:1 binding model and local fit to obtain the association rate constant (k_a) and dissociation rate constant (k_d).

Information of cell-displayed DRP lentiviral plasmid library-1 and 2

Sequences of the oligonucleotides (5' to 3') and reverse primers used for cell-displayed DRP lentiviral plasmid libraries cloning were described as follows. N represents any of the 4 nucleotides (A, G, C and T) and K represents thymidine (T) and guanosine (G). Two cell-displayed DRP lentiviral libraries were constructed by different oligonucleotides which encode different exogenous peptides. Table S1 shows the information of displayed peptides and capacity of different libraries.

Oligonucleotides of cell-displayed DRP lentiviral plasmid library-1:

5'−TCGCTCGCACGCGTTGCCCCCCTTGCNNKNNKNNKNNKNNKTGTNNKNNKNNKNNKNNKTGTNNKNNKNNKNNKN
NKTGCCCCCCTTGCCTGTACAAAGGTGCGCCGGTG−3'

Reverse primers for constructing library-1:

5'−CACCGGCGCACCTTTGTACAGGC−3'

Oligonucleotides of cell-displayed DRP lentiviral plasmid library-2:

5'−TCGCTCGCACGCGTTGCCCCCCTTGCCGTTTTACTCTGAGTNNKNNKNNKNNKNNKTGTNNKNNKNNKNNKNNKCCT
ATTCGGGCTATGTGTAATGATTCTGTTCCGTGCCCCCCTTGCCTGTACAAAGGTGCGCCGGTG−3'

5'−TCGCTCGCACGCGTTGCCCCCCTTGCTTGCTTGAGAATAATNNKNNKNNKNNKNNKTGTNNKNNKNNKNNKNNKAG

TCTTATTTTTCAGTGTAATTTTATTCCGAATTGCCCCCCTTGCCTGTACAAAGGTGCGCCGGTG−3'

5'−TCGCTCGCacgcgtTGCCCCCCTTGCGATCTTCTTAAGCTTNNKNNKNNKNNKNNKTGTNNKNNKNNKNNKNNKACTTC

TATTATTACGTGTTATTCTAATCATACTTGCCCCCCTTGCCTGTACAAAGGTGCGCCGGTG−3'

Reverse primers for constructing library-2:

5'−CACCGGCGCACCTTTGTACAGGC−3'

Table S1 Information of cell-displayed DRP lentiviral plasmid library-1 and 2

Number	Displayed peptides	Capacity
**library-1**	CPPCXXXXXCXXXXXCXXXXXCPPC	3.0 × 10^6 pfu
**library-2**	CPPCRFTLSXXXXXCXXXXXPIRAMCNDSVPCPPC<br>CPPCDLLKLXXXXXCXXXXXTSIITCYSNHTCPPC<br>CPPCLLENNXXXXXCXXXXXSLIFQCNFIPNCPPC	3.36 × 10^6 pfu

Information of primers

Table S2 Sequence of primers

Number	Sequences
**primer 1**	5'−TAGGGATAACAGGGTAATCCGCTAGCGGGTAGGGGAGGCGTTTTC−3'
**primer 2**	5'−TTGTAATCCAGAGGTTGATTGTCGACTCAGGCACCGGGCTTGC−3'
**primer 3**	5'−GGCACTGACAATTCCGTGGT−3'
**primer 4**	5'−AGGGACGTAGCAGAAGGACG−3'
**primer 5**	5'−CCCATTCTATCATCAACGGGTACAA−3'
**primer 6**	5'−CAGCAAGTGGGAAGGTGTAATCC−3'
**primer 7**	5'−CCTTAAGGTTAACTCTAGAGAATTCGGATCCAATATTC−3'
**primer 8**	5'−GGGGGAGGGAGAGGGGCTAG−3'
**primer 9**	5'−TCGCAGGCCCAGCCGGCCATGGCATCCGGCGGAAGCACGCGT−3'
**primer 10**	5'−GTAGTAACGCGGCCGCGCCGCTGCCGCTGTACAG−3'

Information of peptides

Table S3 Sequence and molecular weight of peptides

Number	Sequence	Molecular weight Reduced	Molecular weight Oxidized
**drp1**	H-GCPPCRFTLSPWVSPCSNPHWPIRAMCNDSVPCPPC-NH_2	3955.7480	3949.7020
**drp2**	H-CPPCRFTLSEDNLRCGKWTMPIRAMCNDSVPCPPC-NH_2	3941.7575	3935.7106
**drp3**	H-GCPPCDLLKLLAHPPCYRDWNTSIITCYSNHTCPPC-NH_2	4032.8398	4026.7928
**drp4**	H-GCPPCDLLKLMGETRCQPLELTSIITCYSNHTCPPC-NH_2	3937.8157	3931.7687
**k-1**	H-WCPPCRFTLSEETGECQGTRRPIRAMCNDSVPCPPC-NH_2	4040.7823	4034.7353
**k-2**	H-WCPPCRFTLSPETGECQGTKRPIRAMCNDSVPCPPC-NH_2	3980.7864	3974.7394

Flow cytometry analysis

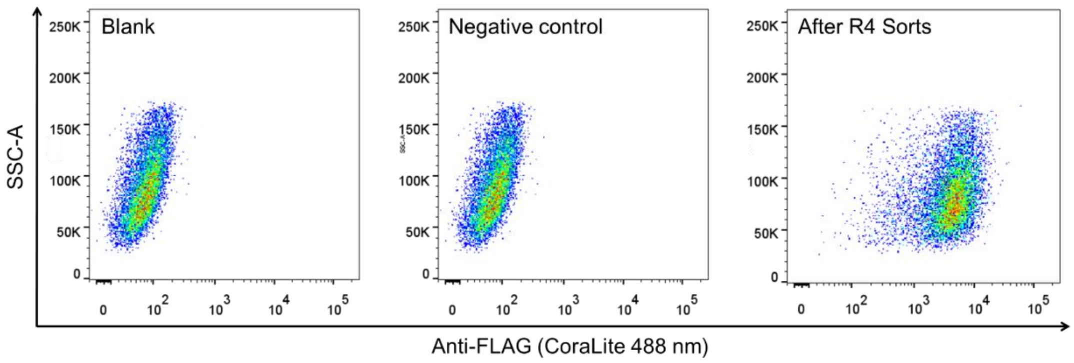

Figure S1. Immunofluorescence flow cytometry analysis of cell-displayed DRP library-1. Blank: cells without the virus infection; Negative control: cells with the infection of virus without the inserted random peptide and FLAG tag; After R4 Sorts: cells amplified from P4 cell population.

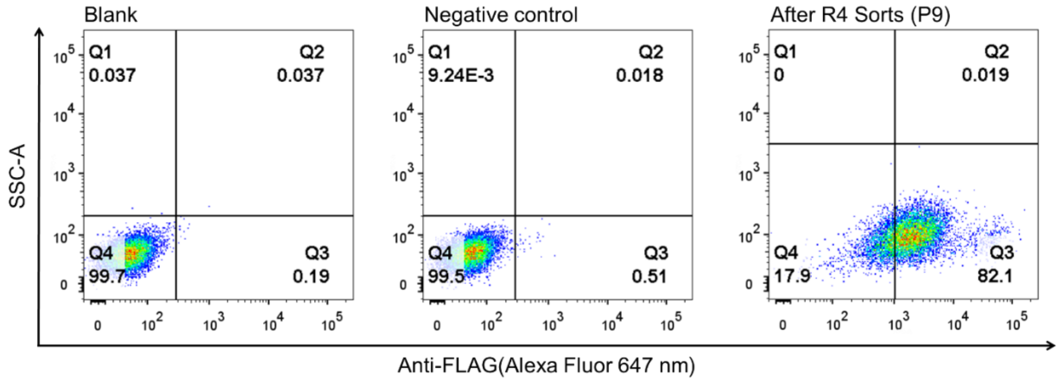

Figure S2. Immunofluorescence flow cytometry analysis of cell-displayed DRP library-2. Blank: cells without the virus infection; Negative control: cells with the infection of virus without the inserted random peptide and FLAG tag; After R4 Sorts (P9): cells amplified from P9 cell population.

Sequencing results

Cell clones	Sequences	Percentage/%
P4-1	CPPCPTQDICLSPNVCITFMACPPC	56.38
	CPPCQLNIMCNHTWCCNNYNSCPPC	8.05
	CPPCQW*TRCRLLLRCVLSDRCPPC	7.90
	CPPC*AERLCSRRWSCMGEASCPPC	7.63
	CPPCRILFTCLIDSNCNILKLCPPC	6.54
	CPPCI*NFVCYIIIFCNILYSCPPC	6.09
	CPPCYFRDSCRIIFWCGFVS*CPPC	5.71
P4-2	CPPCHYPRECIFRNPCSIIQECPPC	27.98
	CPPCVAFLNCNNTHLCTTLKTCPPC	25.89
	CPPCAKFLNCYFCHQCYIHL*CPPC	22.90
	CPPCYINFICLFVMICFYFTNCPPC	14.52
P4-3	CPPCRFTLSCPIRAMCNDSVPCPPC	32.41
	CPPCGSPTGCIPLSNCMSNYTCPPC	19.15
	CPPCERGGRCCREINCYPRVLCPPC	18.08
	CPPCVGFRRCVYMTSCIILRNCPPC	16.19
	CPPCLNISKCYFYSHCNFIYFCPPC	10.51
P4-4	CPPCDLLKLCTSIITCYSNHTCPPC	33.29
	CPPCCFEMLC*IFYSCFTKPNCPPC	31.84
	CPPC*FISCCFNLIFCERLYSCPPC	28.48
P4-5	CPPCGRSLSCSVHMRCLFLTACPPC	11.94
	CPPCPLKMHCNYFNMCSSINTCPPC	10.96
	CPPCSILGPCHFKLNCTHDLVCPPC	10.87
	CPPCSDHFMCNLSHLCTFTYDCPPC	10.76
	CPPCSHYN*CYIHPYCYTNHFCPPC	10.06
	CPPCLNYDLCLSYNLCSRKNFCPPC	9.49
	CPPCRSDFICHCPSFCFLLYNCPPC	8.20
	CPPCYHHFICYIYYYCINNFFCPPC	7.43
P4-6	CPPCRFTLSCPIRAMCNDSVPCPPC	32.15
	CPPCGSPTGCIPLSNCMSNYTCPPC	24.01
	CPPCVGFRRCVYMTSCIILRNCPPC	18.93
	CPPCERGGRCCREINCYPRVLCPPC	13.53
	CPPCLNISKCYFYSHCNFIYFCPPC	11.38
P4-7	CPPCCKLACCKFKCLCPCPTNCPPC	56.33
	CPPCQIVNNCTNLSTCELPNHCPPC	17.89
	CPPCIFPNHCSNYTVCNNAPLCPPC	15.79
P4-8	CPPCGSPTGCIPLSNCMSNYTCPPC	26.53
	CPPCVGFRRCVYMTSCIILRNCPPC	20.36
	CPPCRFTLSCPIRAMCNDSVPCPPC	19.66
	CPPCLNISKCYFYSHCNFIYFCPPC	10.12
P4-9	CPPCPTQDICLSPNVCITFMACPPC	46.62
	CPPCQLNIMCNHTWCCNNYNSCPPC	10.21
	CPPCQW*TRCRLLLRCVLSDRCPPC	9.39
	CPPC*AERLCSRRWSCMGEASCPPC	8.55
	CPPCRILFTCLIDSNCNILKLCPPC	6.16
P4-10	CPPCRPMDRCITSSGCHNYPSCPPC	15.80
	CPPCNNFKLCGNMLSCSSHFICPPC	12.79
	CPPCVKCTFC*R*FVCNSIFLCPPC	12.11
	CPPCENAMPCLRHIFCNFILACPPC	11.97
	CPPCSLFYPCIRRLSCMDYLPCPPC	11.85
	CPPCLSL*ICLTNAVCCLLIHCPPC	11.70
	CPPCEINFICNIGTNCLNFTFCPPC	11.38
	CPPCSSYNFCFINYHCFFYITCPPC	8.32

Figure S3. Deep-sequencing results of 10 randomly-selected cell clones from P4 cell population (* denotes terminator).

No.		Abundance Input	After R4 Sorts	Normalization
M-1	CPPCPLKMHCNYFNMCSSINTCPPC	4	856	214
M-2	CPPCRFTLSCPIRAMCNDSVPCPPC	12	1800	150
M-3	CPPCYHHFICYIYYYCINNFFCPPC	1	136	136
M-4	CPPCRILFTCLIDSNCNILKLCPPC	11	1296	118
M-5	CPPCGSPTGCIPLSNCMSNYTCPPC	11	1132	103
M-6	CPPCDLLKLCTSIITCYSNHTCPPC	15	1162	77
M-7	CPPCVGFRRCVYMTSCIILRNCPPC	12	906	76
M-8	CPPCPTQDICLSPNVCITFMACPPC	146	10320	71
M-9	CPPCLLENNCSLIFQCNFIPNCPPC	2	137	69
M-10	CPPCSILGPCHFKLNCTHDLVCPPC	14	928	66
M-11	CPPCYWSIFCFYSYWCIFYNLCPPC	1	57	57
M-12	CPPCPIYNLCYINHSCPIFPNCPPC	2	17	9

Figure S4. 12 peptides from P4 cell population were randomly-selected to analyze the expression level on the cell surface.

Cell clones	Sequences	Percentage/%
P8-1	CPPCLLENNQAANLCAQVCCSLIFQCNFIPNCPPC	48.76
	CPPCDLLKLNYHHDCFRHSFTSIITCYSNHTCPPC	42.77
P8-2	CPPCDLLKLVCIPACNALDATSIITCYSNHTCPPC	50.56
	CPPCDLLKLSEDSLCSLYSCTSIITCYSNHTCPPC	46.27
P8-3	CPPCDLLKLSQLDRCGGLPVTSIITCYSNHTCPPC	85.89
	CPPCRFTLSSEYSTCNWCLIPIRAMCNDSVPCPPC	3.68
P8-4	CPPCDLLKLPFLLRCTLHSSTSIITCYSNHTCPPC	87.36
	CPPCRFTLSVLSLSCPMPSSPIRAMYNDSVPCPPC	4.46
P8-5	CPPCDLLKLVVCLDCETRHRTSIITCYSNHTCPPC	52.65
	CPPCLLENNHYLRRCTAQVQSLIFQCNFIPNCPPC	16.73
	CPPCDLLKLGSRRYCPVG*RTSIITCYSNHTCPPC	14.96
	CPPCLLENNTRSFNCIDFYLSLIFQCNFIPNCPPC	12.73
P8-6	CPPCLLENNSFELRCKAQDTSLIFQCNFIPNCPPC	54.52
	CPPCLLENNLSIATCSYFAPSLIFQCNFIPNCPPC	45.48
P8-7	CPPCLLENNAVLV*CVARGMSLIFQCNFIPNCPPC	70.59
	CPPCRFTLSPPRIPCKTNTIPIRAMCNDSVPCPPC	19.30
P8-8	CPPCLLENNVRVCTCYRYNSSLIFQCNFIPNCPPC	45.06
	CPPCLLENNMTHSSCPCTVMSLIFQCNFIPNCPPC	36.40
P8-9	CPPCDLLKLTVPQGCVTVQCTSIITCYSNHTCPPC	36.58
	CPPCLLENNSFELRCKAQDTSLIFQCNFIPNCPPC	31.64
	CPPCLLENNLSIATCSYFAPSLIFQCNFIPNCPPC	26.33
P8-10	CPPCDLLKLVPHRGCSGDTVTSIITCYSNHTCPPC	86.78
	CPPCRFTLSNNVDRCNQTSPPIRAMCNDSVPCPPC	6.33
P8-11	CPPCRFTLSANQWTCIMERFPIRAMCNDSVPCPPC	50.50
	CPPCRFTLSSNSSGCRGMVGPIRAMCNDSVPCPPC	42.39
P8-12	CPPCLLENNSFELRCKAQDTSLIFQCNFIPNCPPC	50.67
	CPPCLLENNLSIATCSYFAPSLIFQCNFIPNCPPC	45.60
P8-13	CPPCDLLKLLLETFCGSETATSIITCYSNHTCPPC	93.78
P8-14	CPPCLLENNWTCRICVMRANSLIFQCNFIPNCPPC	94.13
P8-15	CPPCDLLKLLLVLDCNGSP*TSIITCYSNHTCPPC	50.07
	CPPCDLLKLCFNDRCFIDSPTSIITCYSNHTCPPC	46.00

Figure S5. Deep-sequencing results of 15 randomly-selected cell clones from P8 cell population (* denotes terminator).

Sequences	Abundance
CPPCRFTLSQDYLFCGNFYAPIRAMCNDSVPCPPC	8
CPPCRFTLSSCLSRCNSSLVPIRAMCNDSVPCPPC	6
CPPCDLLKLGDVPVCCELSRTSIITCYSNHTCPPC	3
CPPCRFTLSPWVSPCSNPHWPIRAMCNDSVPCPPC	2
CPPCRFTLSTANPLCIRPYPPIRAMCNDSVPCPPC	1
CPPCRFTLSCMKTMCSSFDNPIRAMCNDSVPCPPC	1
CPPCDLLKLPRRYCCLEVGATSIITCYSNHTCPPC	1
CPPCDLLKLLYNSFCPPPPATSIITCYSNHTCPPC	1
CPPCDLLKLHIPAWCEARILTSIITCYSNHTCPPC	1
CPPCDLLKLCYSFPCQYRLATSIITCYSNHTCPPC	1
CPPCRFTLSNEYFDCSLYFAPIRAMCNDSVPCPPC	1

Figure S6. Sanger sequencing results of 26 randomly-selected cell clones from P9 cell population.

Chromatograms of peptides

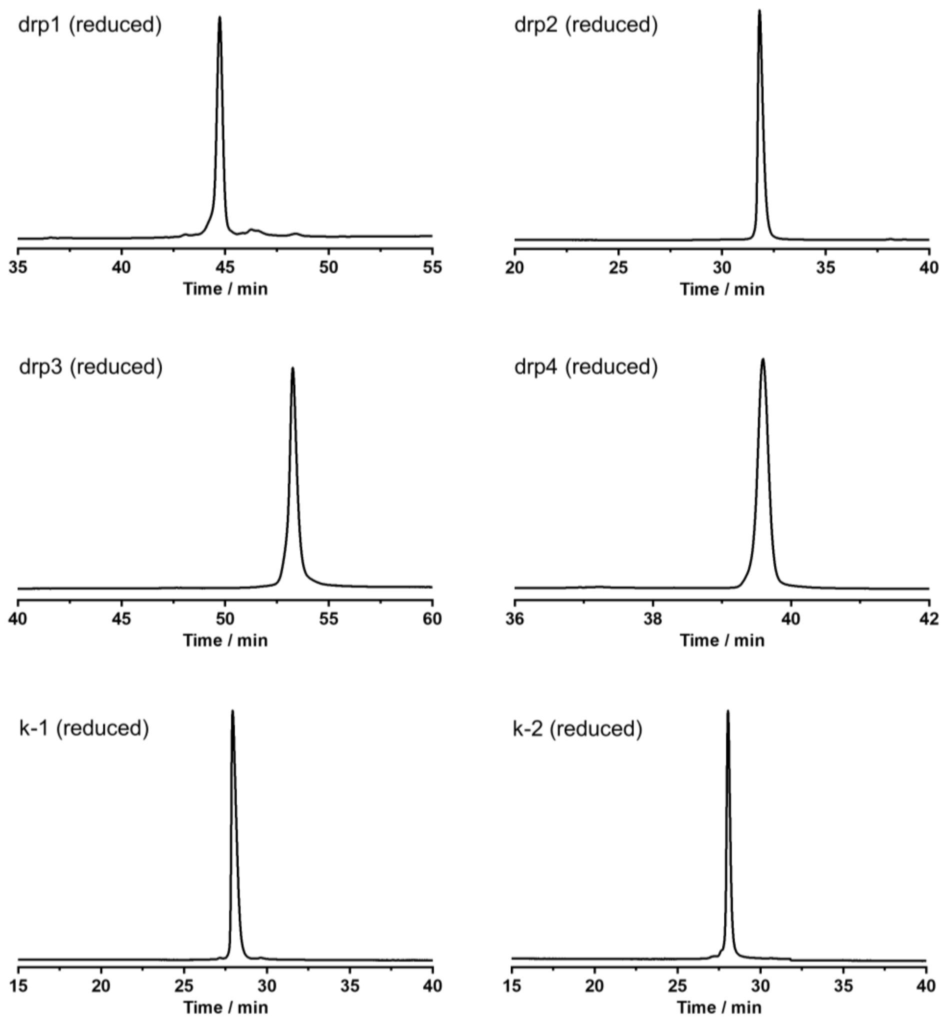

Figure S7. Chromatograms showing peptides **drp1–4 and k-1, k-2** (purity: >95%).

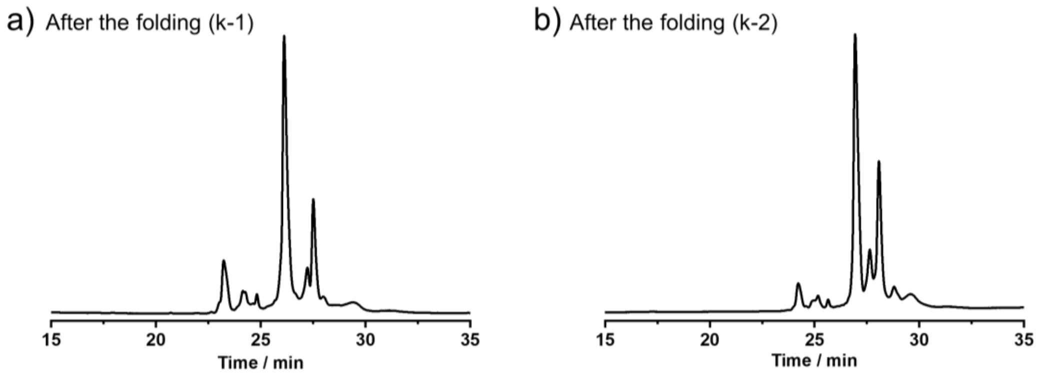

Figure S8. a) Chromatograms showing the products formed after the oxidation of **k-1**. b) Chromatograms showing the products formed after the oxidation of **k-2**.

Mass spectrum of peptides

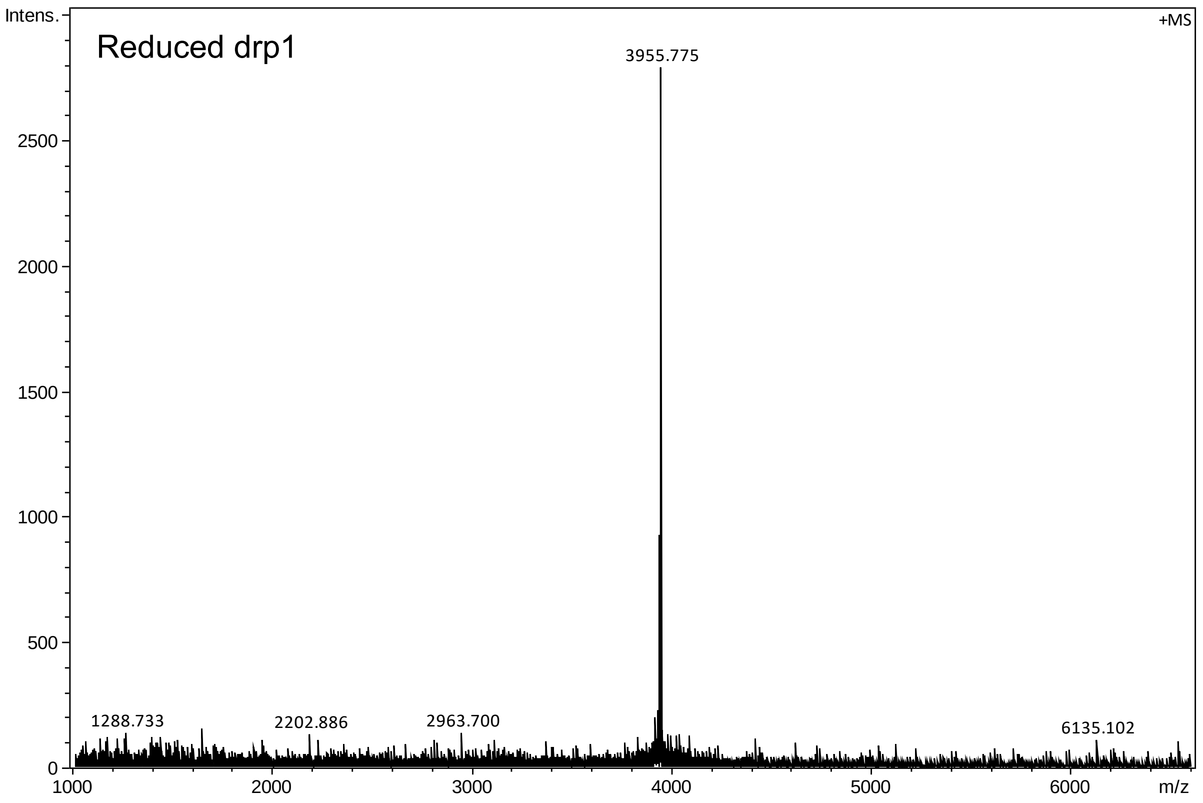

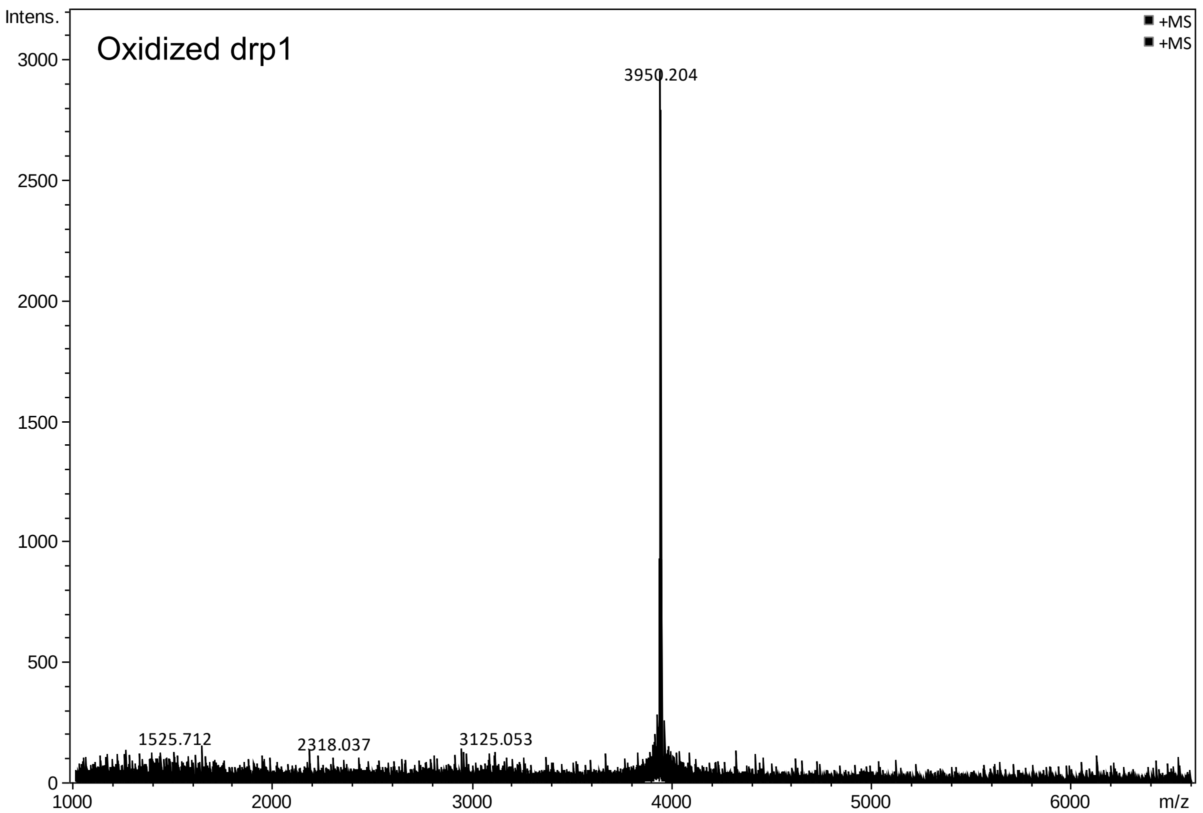

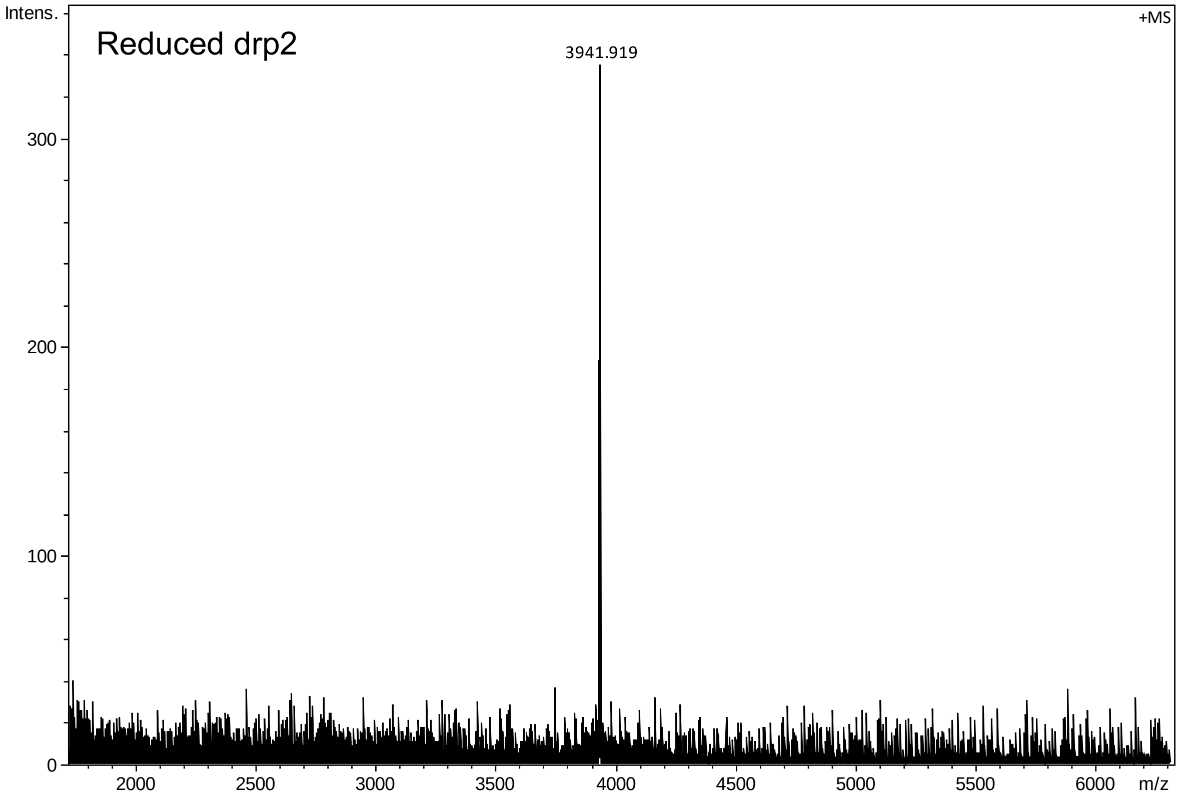
Intens.
Reduced drp2
+MS
3941.919
300
200
100
0
2000
2500
3000
3500
4000
4500
5000
5500
6000
m/z

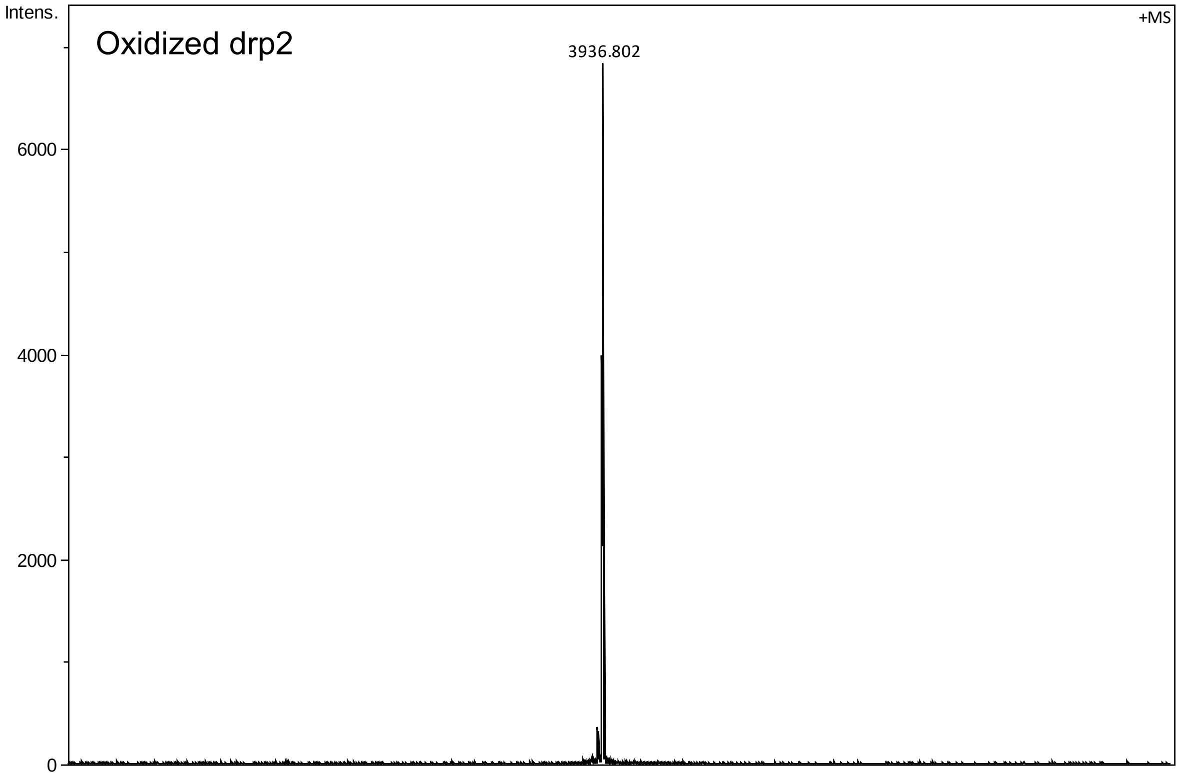
Intens.
Oxidized drp2
+MS
3936.802
6000
4000
2000
0

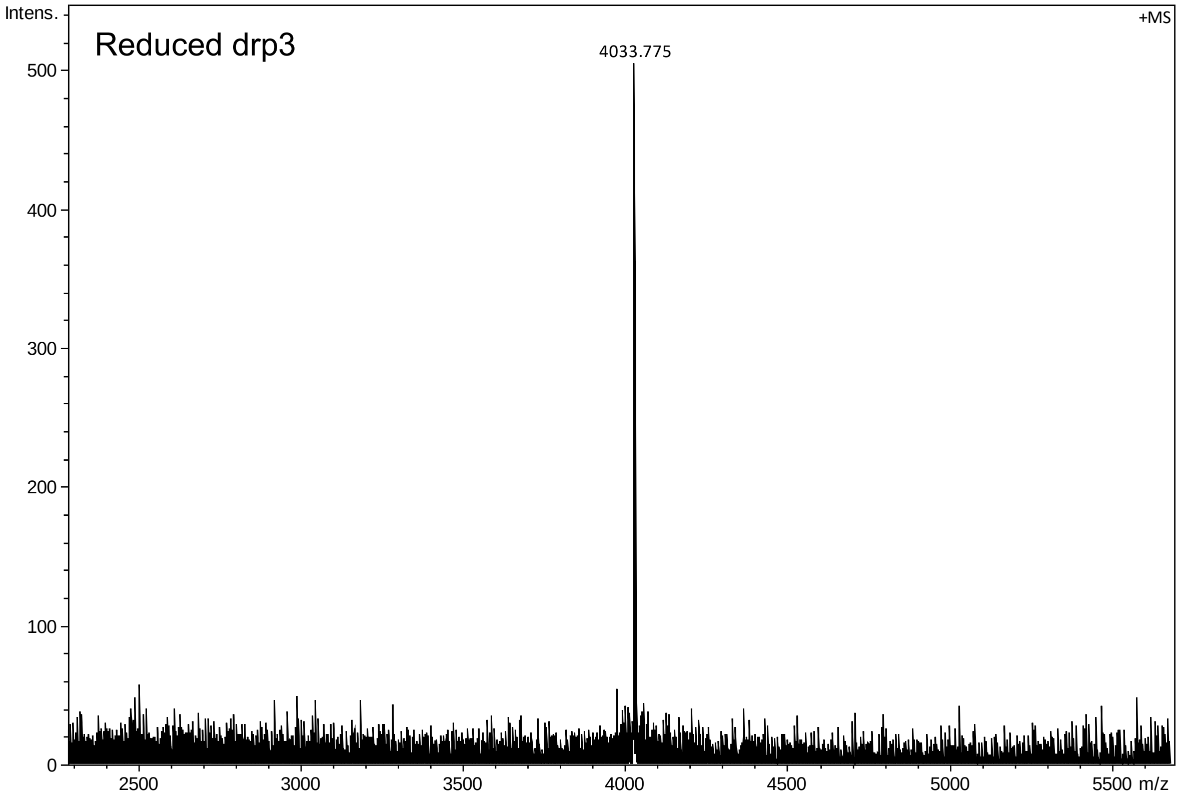
Intens.
Reduced drp3
+MS
4033.775
500
400
300
200
100
0
2500
3000
3500
4000
4500
5000
5500 m/z

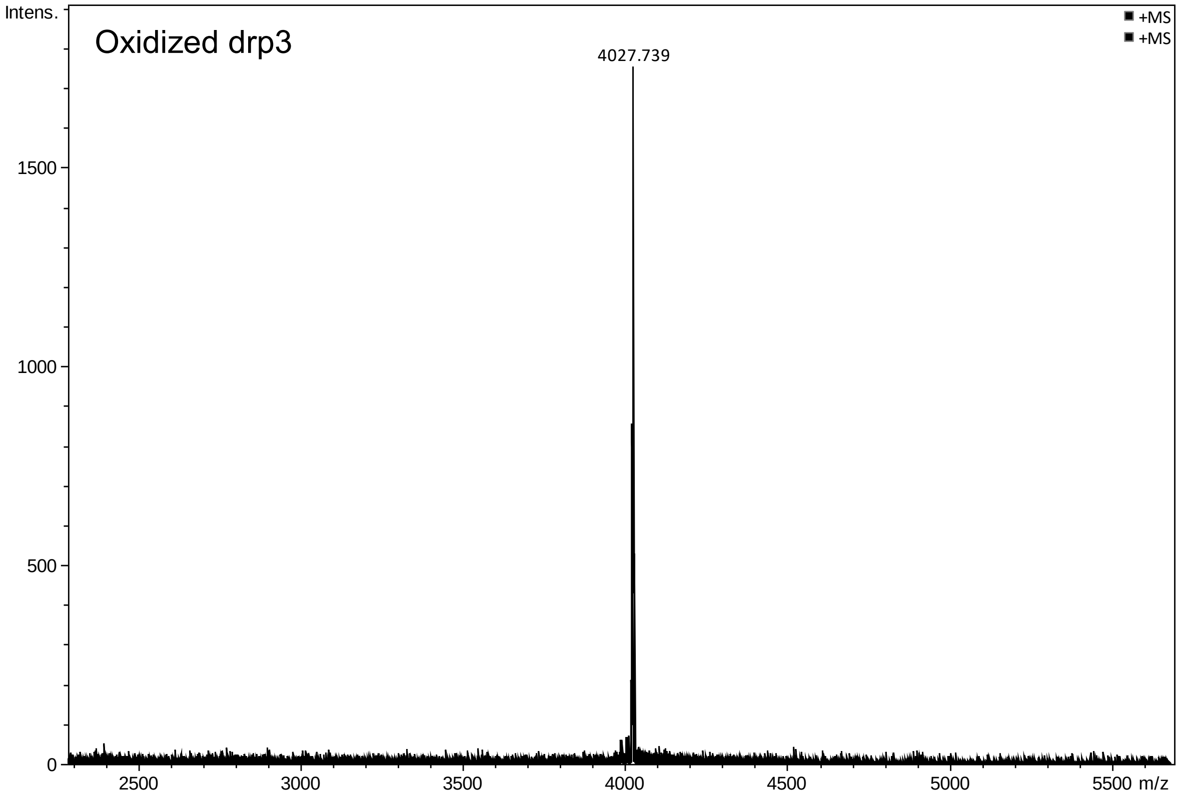
Intens.
Oxidized drp3
+MS
+MS
4027.739
1500
1000
500
0
2500
3000
3500
4000
4500
5000
5500 m/z

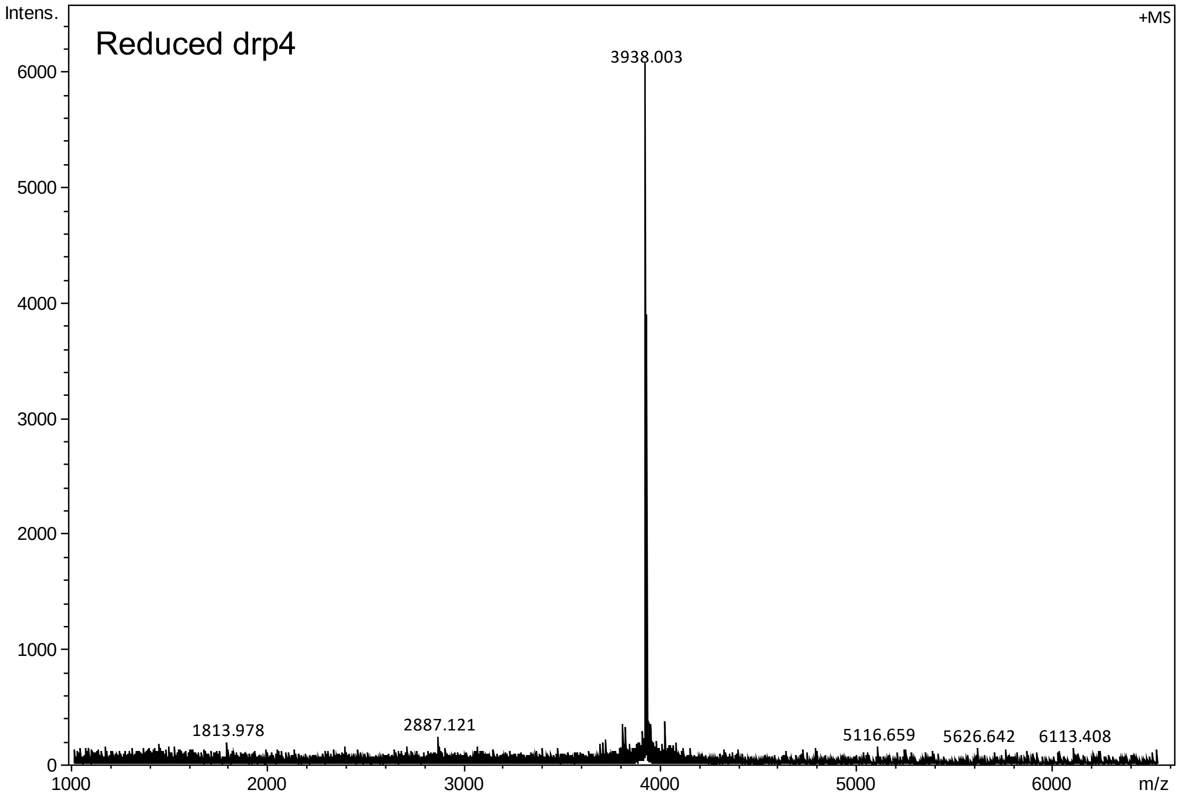
Intens.
Reduced drp4
+MS
3938.003
1813.978
2887.121
5116.659
5626.642
6113.408
6000
5000
4000
3000
2000
1000
0
1000
2000
3000
4000
5000
6000
m/z

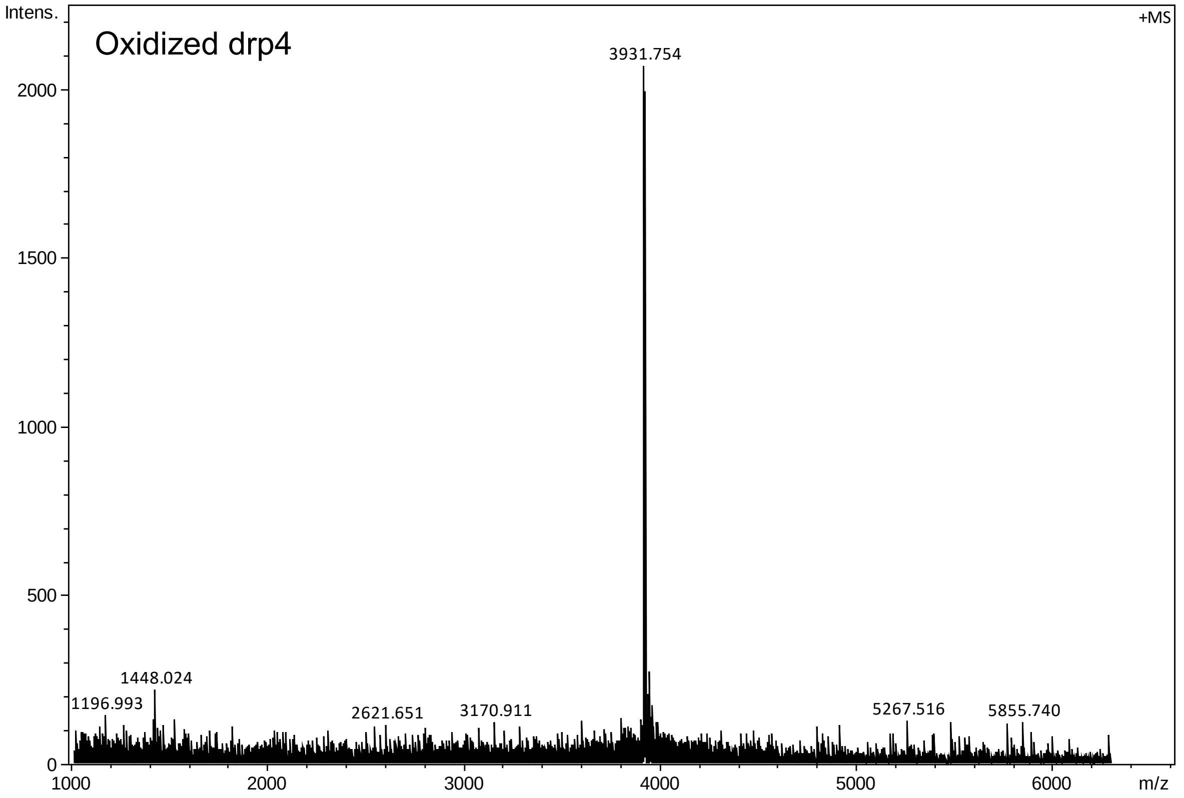
Intens.
Oxidized drp4
+MS
3931.754
1448.024
1196.993
2621.651
3170.911
5267.516
5855.740
2000
1500
1000
500
0
1000
2000
3000
4000
5000
6000
m/z

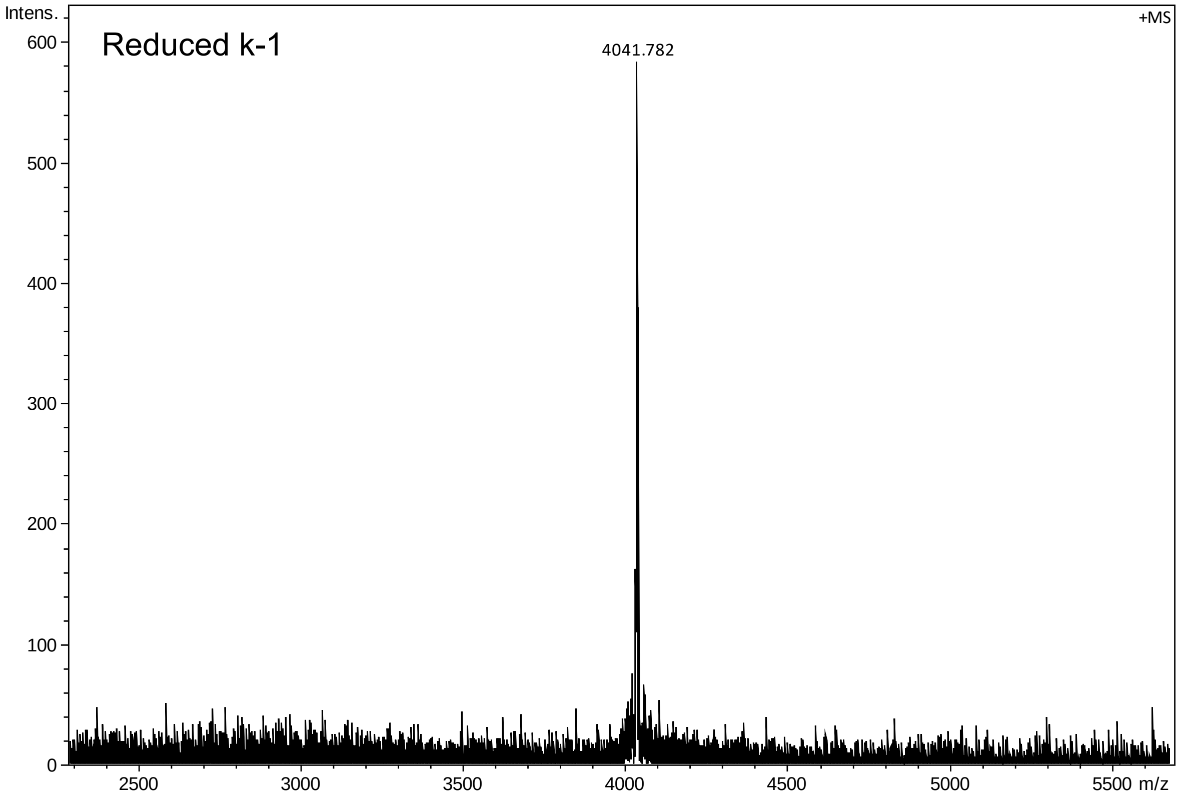
Intens.
Reduced k-1
+MS
4041.782
600
500
400
300
200
100
0
2500
3000
3500
4000
4500
5000
5500 m/z

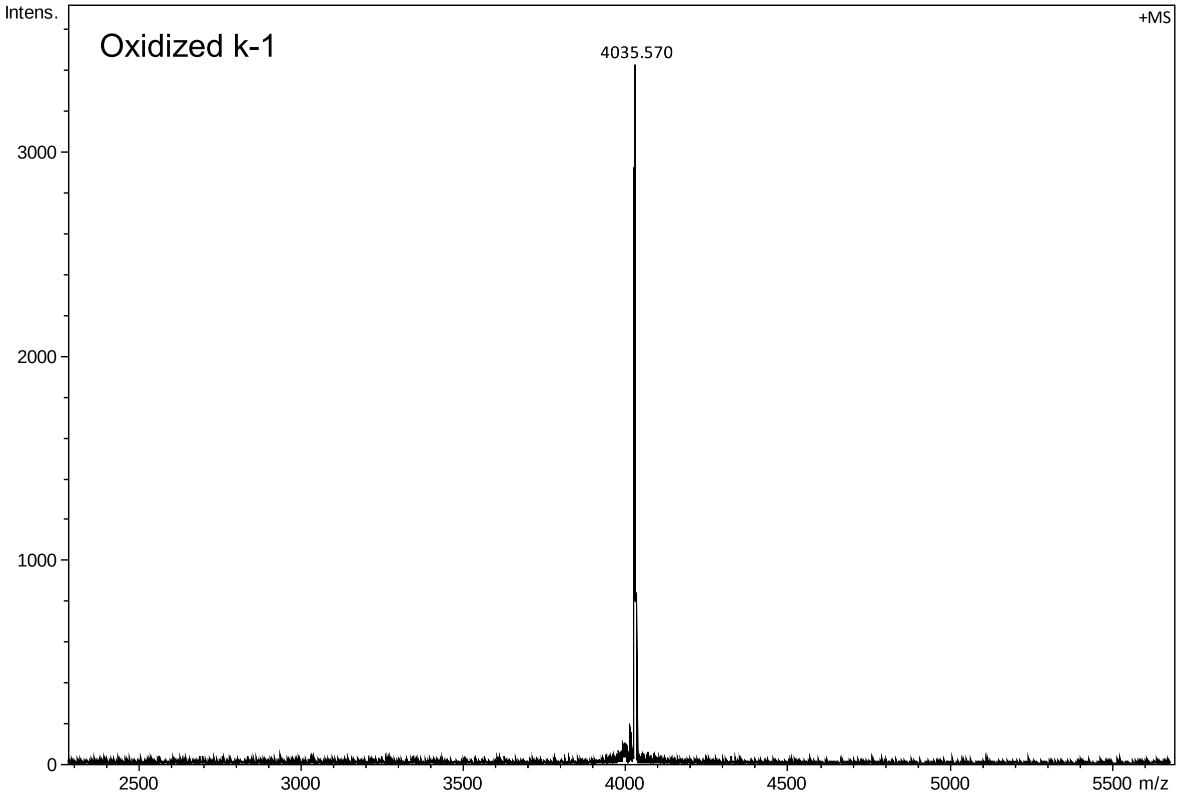
Intens.
Oxidized k-1
+MS
4035.570
3000
2000
1000
0
2500
3000
3500
4000
4500
5000
5500 m/z

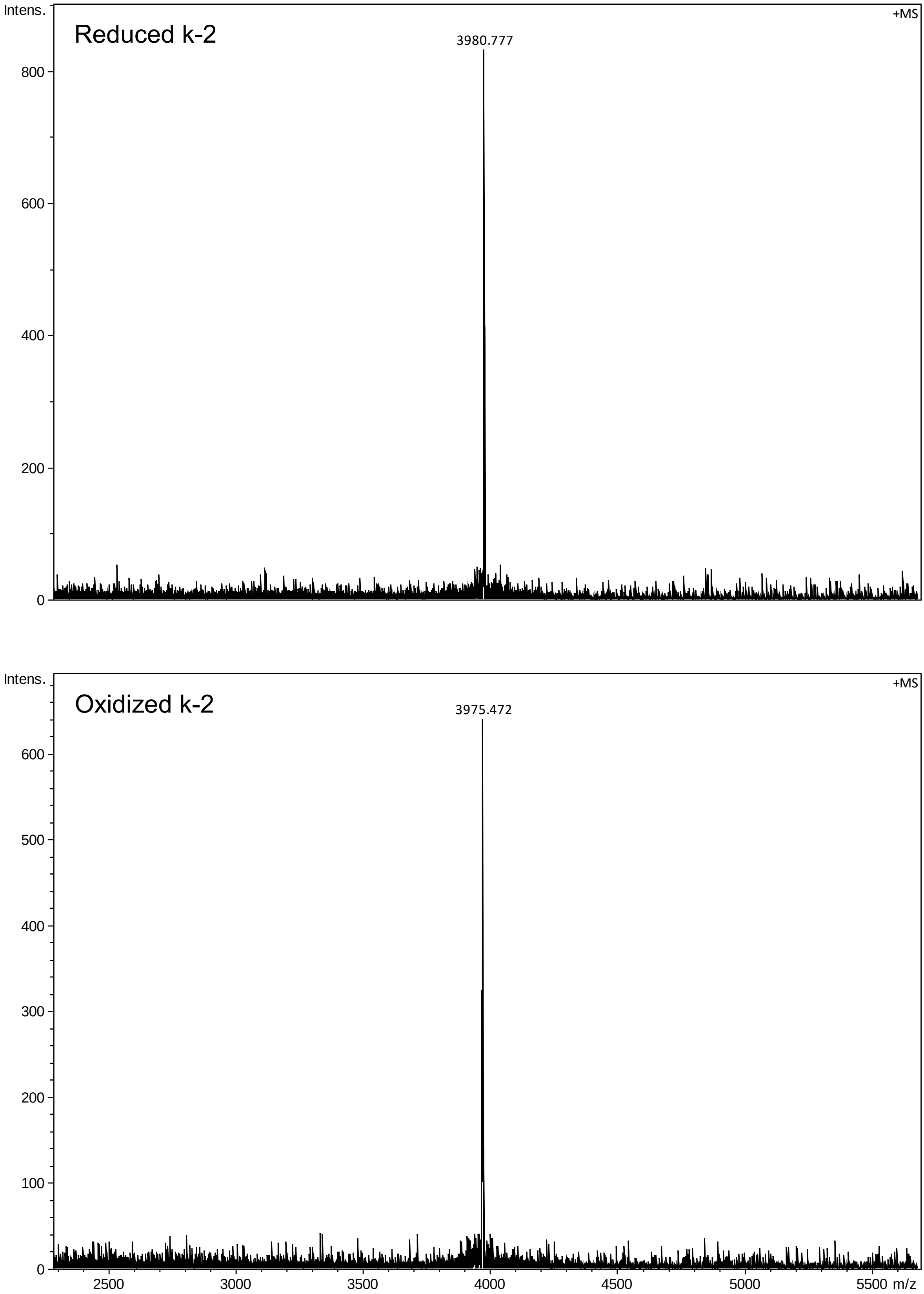

Figure S9. Mass spectrum of peptides **drp1-4, k-1, k-2** (reduced and oxidized).

NMR characterization of oxidized drp1.

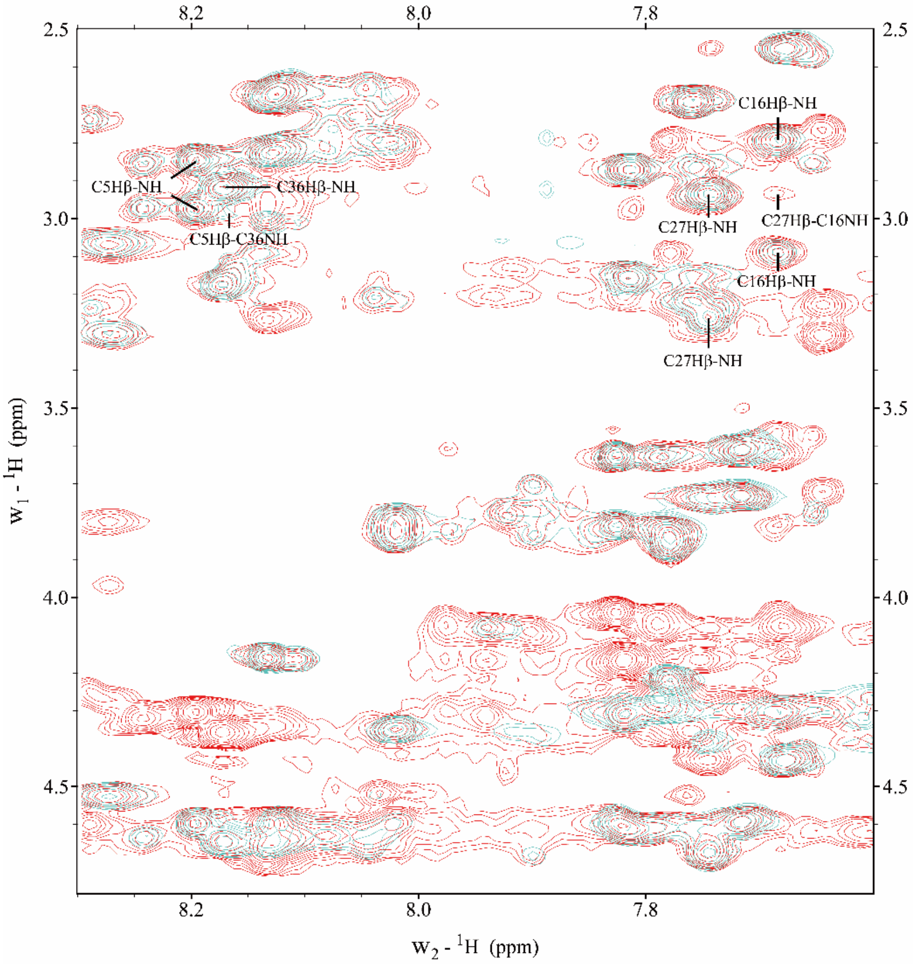

Figure S10. (a) NMR Characterization of oxidized **drp1**. 2D 1H-1H NOESY (red) and TOCSY (blue) spectra of oxidized **drp1** in ACN/H_2O (80%/20%, v/v). Only local regions were plotted to show cross peaks of cysteine residues (disulfide connectivity: Cys 5-Cys 36, Cys 16-Cys 27) and cysteine-related assignments were labeled.

Characterization and affinity experiments of the major oxidized product of k-1 and k-2

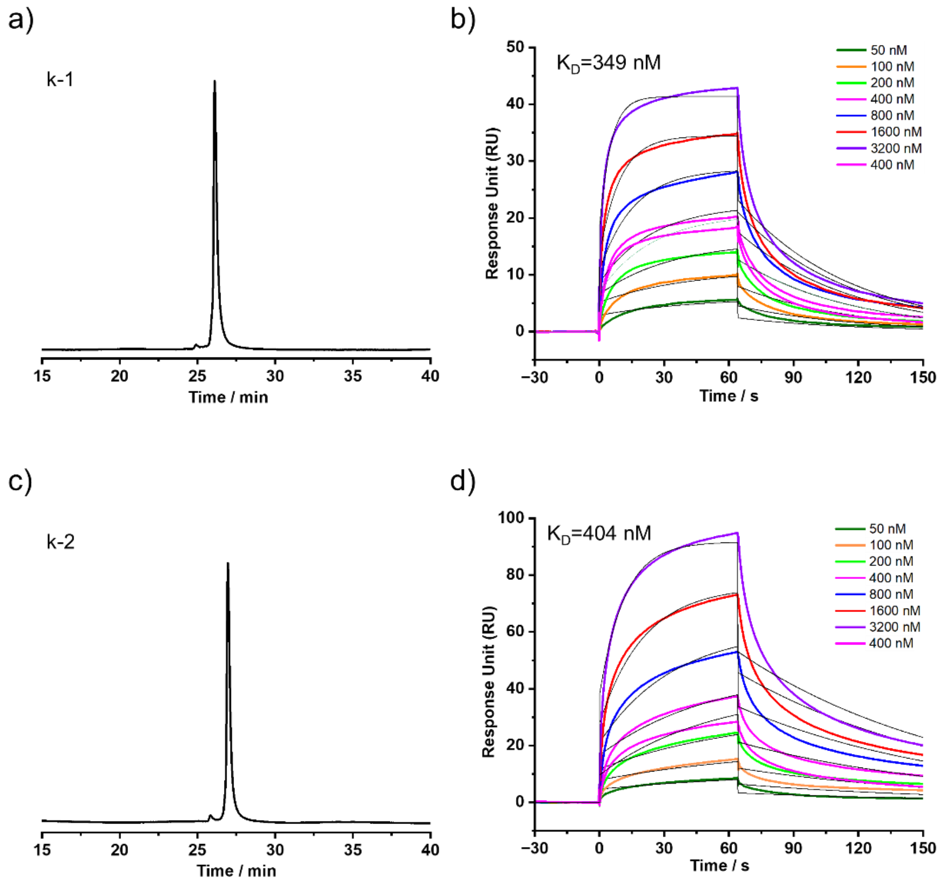

Figure S11. (a) The oxidized **k-1** used for SPR assays was purified using HPLC to a purity of >95%. (b) SPR sensorgrams showing the binding of Keap1 with the oxidized **k-1** in a concentration-dependent manner. (c) The oxidized **k-2** used for SPR assays was purified using HPLC to a purity of >95%. (d) SPR sensorgrams showing the binding of Keap1 with the oxidized **k-2** in a concentration-dependent manner.

References

1. W. Barczak, W. Suchorska, B. Rubis and K. Kulcenty, *Mol Biotechnol*, 2015, **57**, 195-200.
2. R. H. Kutner, X. Y. Zhang and J. Reiser, *Nat Protoc*, 2009, **4**, 495-505.
3. I. Rentero Rebollo, M. Sabisz, V. Baeriswyl and C. Heinis, *Nucleic Acids Res*, 2014, **42**, e169.
4. S. Lu, Y. Wu, J. Li, X. Meng, C. Hu, Y. Zhao and C. Wu, *Journal of the American Chemical Society*, 2020, **142**, 16285-16291.
5. F. Delaglio, S. Grzesiek, G. W. Vuister, G. Zhu, J. Pfeifer and A. Bax, *J. biomol. NMR*, 1995, **6**, 277-293.
6. Y. Shen and A. Bax, *J. biomol. NMR*, 2013, **56**, 227-241.
7. W. Rieping, M. Habeck, B. Bardiaux, A. Bernard, T. E. Malliavin and M. Nilges, *Bioinformatics*, 2007, **23**, 381-382.
8. A. T. Brünger, P. D. Adams, G. M. Clore, W. L. DeLano, P. Gros, R. W. Grosse-Kunstleve, J.-S. Jiang, J. Kuszewski, M. Nilges and N. S. J. A. C. S. D. B. C. Pannu, *Acta Crystallogr. Section D Biol. Crystallogr.*, 1998, **54**, 905-921.
9. R. A. Laskowski, J. A. C. Rullmann, M. W. MacArthur, R. Kaptein and J. M. J. J. o. b. N. Thornton, *J. biomol. NMR*, 1996, **8**, 477-486.
